# FlowAtlas: an interactive tool for high-dimensional immunophenotyping analysis bridging FlowJo with computational tools in Julia

**DOI:** 10.3389/fimmu.2024.1425488

**Published:** 2024-07-17

**Authors:** Valerie Coppard, Grisha Szep, Zoya Georgieva, Sarah K. Howlett, Lorna B. Jarvis, Daniel B. Rainbow, Ondrej Suchanek, Edward J. Needham, Hani S. Mousa, David K. Menon, Felix Feyertag, Krishnaa T. Mahbubani, Kourosh Saeb-Parsy, Joanne L. Jones

**Affiliations:** ^1^ Department of Clinical Neurosciences, University of Cambridge, Cambridge, United Kingdom; ^2^ Randall Centre for Cell & Molecular Biophysics, King’s College London, London, United Kingdom; ^3^ Department of Medicine, University of Cambridge, Cambridge, United Kingdom; ^4^ Department of Anaesthesia, University of Cambridge, Cambridge, United Kingdom; ^5^ Independent Researcher, Oxford, United Kingdom; ^6^ Department of Surgery, University of Cambridge, Cambridge, United Kingdom; ^7^ Collaborative Biorepository for Translational Medicine (CBTM), Cambridge NIHR Biomedical Research Centre, Cambridge, United Kingdom

**Keywords:** flow cytometry analysis, high-dimensional cytometry, dimensionality reduction, immunophenotyping, spectral flow cytometry, Julia programming language

## Abstract

As the dimensionality, throughput and complexity of cytometry data increases, so does the demand for user-friendly, interactive analysis tools that leverage high-performance machine learning frameworks. Here we introduce FlowAtlas: an interactive web application that enables dimensionality reduction of cytometry data without down-sampling and that is compatible with datasets stained with non-identical panels. FlowAtlas bridges the user-friendly environment of FlowJo and computational tools in Julia developed by the scientific machine learning community, eliminating the need for coding and bioinformatics expertise. New population discovery and detection of rare populations in FlowAtlas is intuitive and rapid. We demonstrate the capabilities of FlowAtlas using a human multi-tissue, multi-donor immune cell dataset, highlighting key immunological findings. FlowAtlas is available at https://github.com/gszep/FlowAtlas.jl.git.

## 1 Introduction

Rapid advancements in flow and mass cytometry have brought about a new era of high-dimensional cell phenotyping. However, traditional gating methods fail to provide an adequate overview of all possible marker combinations, making them insufficient for analyzing such complex datasets. Instead, high-dimensional data is typically visualized by embedding it onto a 2D-map, where the relative distance between data points (events) reflects their phenotypic similarities. In addition to dimensionality reduction (DR), algorithms can automatically identify subpopulations of events with shared characteristics, assigning them into clusters. These clusters can then be projected onto the DR data embedding, enabling users to simultaneously view all populations and parameters within their dataset, assign identities to cell clusters, and discover novel cell populations. Several algorithms have been developed for non-linear DR, including tSNE (1) and UMAP (2). One of the most widely used tools for automatic population clustering is FlowSOM, a self-organizing map (SOM)-based algorithm (3).

DR and cell population clustering algorithms have gradually been integrated into popular analysis platforms such as FCS express and FlowJo, either as core features or add-on plugins. However these can lack downstream interactivity with the DR data and, typically require substantial down-sampling, where only a small portion of the data is selected for analysis to reduce the computational burden. Unfortunately this risks loss of rare cell populations. Furthermore these packages do not support the integration of datasets acquired using different cytometry panels. In contrast, computational pipelines built in scripting languages such as R or Python require significant coding literacy, so hampering their adoption by the wider biomedical community. As data complexity increases and open data access becomes the gold standard there is a growing need for powerful computational tools that do not require coding expertise, can process large datasets, and enable data integration.

Here we introduce FlowAtlas — a free-access, graphical data analysis environment that aims to overcome the limitations of current tools. We chose to write FlowAtlas in Julia (4), a programming language created for high-performance scientific computing and machine learning applications. This gave us access to some of the fastest algorithms available today (4, 5). First, we provide an overview of FlowAtlas’s design and performance, followed by a step-by-step instruction guide for a typical analysis workflow. Using a novel, human flow cytometry dataset, consisting of immune cells extracted from tissues of five deceased organ donors and immunophenotyped using three different antibody panels, we then showcase how FlowAtlas can be used to rapidly and intuitively explore complex data. Then, using publicly available datasets, we demonstrate its ability detect rare cell subsets, and to process data obtained on different cytometry platforms. Finally, we discuss the technical prerequisites needed for robust data analysis in FlowAtlas.

## 2 Results

### 2.1 FlowAtlas design and performance

#### 2.1.1 FlowAtlas integrates with FlowJo: overview

**Figure 1:**
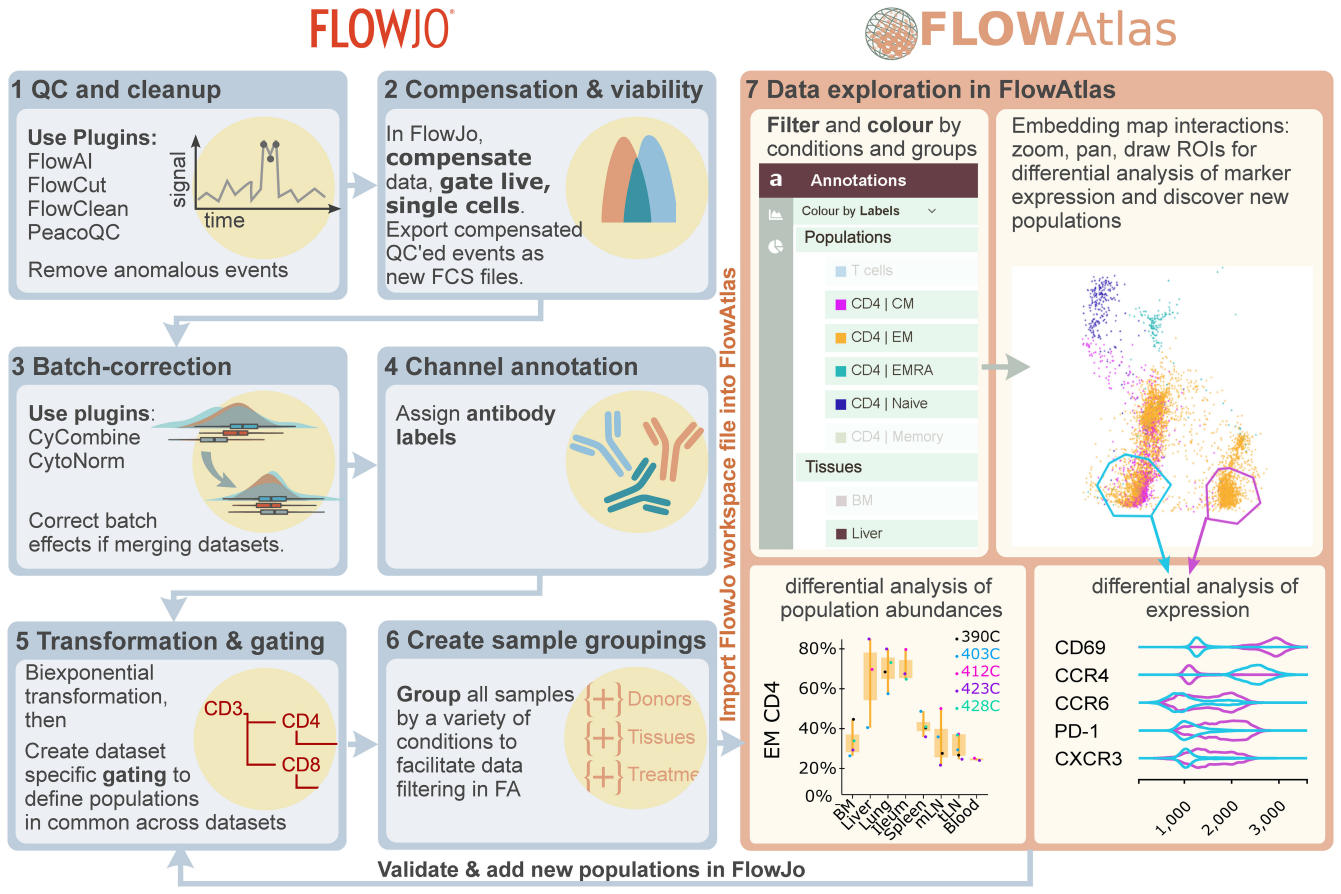
Overview of FlowAtlas workflow with FlowJo. Step 1: Removal of anomalous events using FlowJo plugins. Step 2: Compensation and export of new clean FCS files. Step 3: Batch correction (if required). Steps 4-6: Workspace preparation in FlowJo including resolution of channel naming discrepancies, bi-exponential transformation of all parameters and gating user-defined populations, and sample grouping. Step 7: Importing the workspace into FlowAtlas triggers automatic panel merge, embedding calculation and launches the interactive web interface. Embedded events can be re-coloured and filtered by conditions and groups defined in Step 6. ROIs can be drawn in the embedding, generating violin plots of marker expression. Box plots can be generated to show frequencies of selected populations and conditions. Novel populations identified in FlowAtlas can be validated and annotated in FlowJo. The updated workspace file can then be re-opened in FlowAtlas to import the new annotations. FJ, FlowJo. FA, FlowAtlas.

A major barrier to complex cytometry data exploration for many biologists is the need for coding and bioinformatics expertise. We designed FlowAtlas to be an open source, fully graphical, interactive high-dimensional data exploration tool that does not rely on command-line input or coding literacy. FlowAtlas links the familiar FlowJo workflow with a high-performance machine learning framework enabling rapid computation of millions of high-dimensional events without the need for down-sampling (**Figure 1**).

FlowAtlas reads user-defined settings from FlowJo, including channel names, gate names, sample group names, and the scaling of each individual fluorescence parameter, which is important in discerning positive and negative populations, and therefore in performing DR and clustering analysis. The resultant DR embedding is highly interactive. Users can zoom in to examine deeper cluster structures, apply coloring and filtering to embedded events based on custom conditions, generate frequency statistics, and draw regions of interest (ROIs) to perform comparative analyses of marker expression using violin plots. As individual files are not merged (concatenated), they remain identifiable in the embedding, ensuring that users can see the relative contribution of each sample to trends in their data.

Data exploration happens in an iterative, user-guided discovery loop with FlowJo: traditional FlowJo gating strategies provide the initial annotation of main cell populations, experimental conditions, and sample groupings. The user then switches to FlowAtlas to discover new subpopulations in the interactive embedding, periodically returning to FlowJo to add the new population annotations as they are discovered in FlowAtlas. Analysis does not require any command-line input and is intuitive, similar to zooming in on a geographical map and gradually filling in its features as they come into view.

#### 2.1.2 Compared to other tools, FlowAtlas enables rapid dimensionality reduction without data down-sampling

Existing DR and clustering tools handle large datasets by randomly selecting a subset of the data to reduce computation time (known as down-sampling). This may result in the loss of rare cell populations. We eliminated the need for down-sampling and enabled visual exploration of many millions of cells by utilizing methods within the GigaSOM.jl library in Julia programming language (6). Specifically, the EmbedSOM algorithm from the GigaSOM.jl library performs DR and clustering more efficiently compared to other tools. The developers of EmbedSOM have demonstrated in their benchmarking paper (7) a 10-30-fold reduction in the computational time requirements compared to other popular DR algorithms including UMAP and tSNE. Additionally, EmbedSOM improves clustering performance over the FlowSOM R package (8), utilized by most open-source analysis workflows and commercial software platforms including FlowJo and Cytobank (9). This efficiency was a key reason for using the EmbedSOM algorithm as a building block for FlowAtlas. To handle the challenge of displaying a large number of events on a 2D map without overcrowding, we used tools from the interactive web libraries OpenLayers (10) and D3.js (11), which enable zooming, tiling, and panning of the DR data.

We compared the computational performance of FlowAtlas to two alternative tools for DR that also do not require command-line input under real-life conditions on a laboratory laptop with the following configuration: 64-bit Windows OS, 32GB RAM, 8^th^ generation core i7-8750H processor, 2.20 GHz. Example graphical outputs from DR with each tool are shown in **Supplementary Figure S1**. For this testing, we used a novel tissue-derived immune cell conventional flow cytometry dataset. It consists of 3.88 million total live single cell events (32 FCS files, 19 fluorescence parameters), and samples are stained with 3 different panels (A, B and C). Donor characteristics, panels and antibodies used are shown in **Supplementary Tables S1**–**S3**.

DR of samples stained with panel C (2.32 million events) in FlowJo (v10.8.1) using the inbuilt tSNE function took 49 min. In FCS Express (v7.18.0025), the same samples were processed in 125 min. The full dataset could not be subjected to DR on these platforms because samples stained with different panels cannot be combined. When analyzed as individual files or groups of files (combined by panel), FlowJo tSNE processed the full dataset of 3.88 million events in 6 hours. We did not attempt the same procedure in FCS Express, but it was expected to exceed 125 min required for DR of panel C samples. By contrast, our full dataset (3.88 million events) was processed in FlowAtlas in 18 min (**Table 1**).

**Table 1:** CPU usage and time required by FlowAtlas, FlowJo, and FCS Express to perform dimensionality reduction and/or clustering on a laptop with Windows OS, 32GB RAM, i7-8750H CPU 2.20GHz processor.

Samples	Events (millions)	Dimensionality reduction performance comparison					
		FlowAtlas EmbedSOM		FlowJo tSNE		FCS Express tSNE	
		Time	% CPU	Time	% CPU	Time	% CPU
1 FCS file	0.38	2 min	8.3	7min15s	90	9min30s	100
3 FCS files	2.32	9 min30s	8.5	49 min	90	125 min^a^	100
Full dataset	3.88	18 min	8.5	Up to 6h^†^	100	NR^a b^	NR
Samples		Clustering performance comparison					
		FlowAtlas EmbedSOM		FlowJo EmbedSOM		Cytobank FlowSOM	
		Events (millions)	Time	Events (millions)	Time	Events (millions)	Time
3 FCS spectral files		0.449	2min30s	0.449	5min30s	0.421^c^	12 min
Down-sampling		no		no		yes	

FlowJo version 10.8.1 using its native tSNE tool; FCS Express version 7.18.0025. opt-tSNE settings in both platforms: all fluorescence channels, perplexity 30, iterations 1000, learning rate (eta): automatic; KNN algorithm: ANNOY, with Barnes-hut approximation (=0.5). Times represent best results from 2-3 independent attempts. NR= not run. ^a^Software became unresponsive on 2 of 3 trials. ^b^Different panels cannot be merged so multiple embeddings are produced. ^c^Downsampling required. Computation time for clustering of the indicated number of events from a publicly available spectral dataset in FlowAtlas, FlowJo, and Cytobank. The dataset is from Cytobank experiment number 191382. FlowSOM settings: FlowSOM-on-viSNE, consensus clustering, 23 clustering parameters, without normalization, 20 metaclusters and 100 clusters, seed 770593711. Time in Cytobank excludes the DR step.

As mentioned above, FlowAtlas uses the highly efficient EmbedSOM algorithm, which performs both DR and clustering. Therefore, we also compared the performance of FlowAtlas against two other non-command line clustering tools: the FlowSOM algorithm implemented in the popular subscription-based cloud analysis platform Cytobank; and the EmbedSOM algorithm (v2.1.7) implemented as a FlowJo plugin. For this test, we utilized a spectral cytometry dataset of whole human blood, which is publicly available as a demonstration experiment in Cytobank repository (12). This dataset contains whole peripheral blood samples in 3 FCS files (23 fluorescence parameters, 512,000 events). The published data were already fully unmixed and compensated. Prior to analysis, we excluded debris based on scatter parameters, leaving 449,488 events. In FlowJo (v10.8.1), we recreated the basic gating strategy from the demonstration analysis in Cytobank to identify major cell populations including granulocytes, B-cells, T-cells, and NK cells (**Supplementary Figure S2**). We then subjected the total live single cell events to DR and clustering in FlowAtlas, according to the procedure described in the next section (“Recommended FlowAtlas workflow”). In parallel, we replicated the demonstrated DR analysis in Cytobank, which recommended down-sampling to 420,000 events by equal random sampling (actual number of sampled events= 421,669). Clustering in Cytobank was executed in 12 minutes excluding the time required for prior dimensionality reduction. Finally, we subjected the same cleaned FCS files to EmbedSOM clustering in FlowJo (v10.8.1, EmbedSOM v2.1.7). Computation took 5min 30s and, as expected, it created three maps with different geometry (one per file, since files were not concatenated prior to analysis). The resulting maps had limited interactivity, e.g. drawing gates directly on the map and then examining them by traditional scatter plots. Computation in FlowAtlas took only 2.5min, including embedding time, and as shown later, it enabled us to interact with the data and discover rare cell populations rapidly (see “Demonstrating the utility of FlowAtlas”).

Finally, we stress tested FlowAtlas to confirm that it can perform rapid embedding of very large datasets on a personal computer. We incrementally tested different dataset sizes up to 46 million events and 25 parameters, which embedded in 113 min (**Supplementary Figure S3**). In our hands, the largest dataset committed 41 GB (of 64GB available) RAM, and it could not be processed on an older machine (16 GB RAM). Therefore, higher RAM capability was essential for processing complex datasets, but it still fell well within the capabilities of currently available personal computers. To our knowledge, no other platform is currently equipped to handle cytometry data of this size and complexity without down-sampling. Details and a video demonstration of exploring this large dataset in real time are provided in Methods Section 4.7.

To summarize, we have demonstrated that FlowAtlas rapidly processes large datasets without down-sampling and without the need for specialized computing equipment. Next, we outline the step-by-step procedure we would recommend for analysis in FlowAtlas.

### 2.2 Recommended FlowAtlas workflow: iterative interactive cell population discovery concurrently with FlowJo

A typical analysis workflow using FlowAtlas concurrently with FlowJo is described in **Figure 1**.

STEP 1: As a first step in any analysis, we recommend to quality control raw FCS files and remove anomalous events using dedicated data cleanup tools such as FlowAI (13), FlowCut (14), FlowClean (15), or PeacoQC (16), all of which are now available as FlowJo plugins. For our dataset, we used FlowAI since this was the only data cleanup tool implemented as a FlowJo plugin at the time of our data analysis.

STEP 2: The compensation accuracy for each file is verified and a population of clean live, single cells is gated and exported as new FCS files. Different files may require different compensation matrices. Therefore, when exporting, only compensated fluorescence channels should be selected. This step ensures that the compensation matrix becomes hard-coded in the new FCS files and is accessible to FlowAtlas.

STEP 3: If merging datasets from different experiments or instruments is required, the user will most likely observe batch effects unless measures such as instrument cross-calibration, longitudinal instrument performance normalization and inter-experiment controls were put in place. Removal of batch effects in the absence of inter-run controls that can clearly reveal technical variability is challenging. Before proceeding to FlowAtlas, we recommend batch-correcting the data using dedicated tools such as cyCombine (17) or CytoNorm (18). Both are available as FlowJo plugins. Save batch-corrected files as new FCS files.

STEP 4: Following pre-processing steps 1-3, the new files are annotated in FlowJo. The dataset is opened in a new FlowJo workspace and antibody labels are assigned to fluorescence channels. In our example dataset, the PE channel was used either for FOXP3 or IgM, and CD4 was assigned to either BUV661 or BUV805 (see **Supplementary Table S2**). Therefore, we labelled the PE channel in all panels as FOXP3-IgM and labelled both BUV661 and BUV805 as CD4. Resolving naming discrepancies between channels of non-identical panels is critical because, to perform panel merging, FlowAtlas uses these user-specified channel labels. FlowAtlas defaults to native fluorescence detector names when labels are not provided, which will prevent the panel merge.

**Figure 2:**
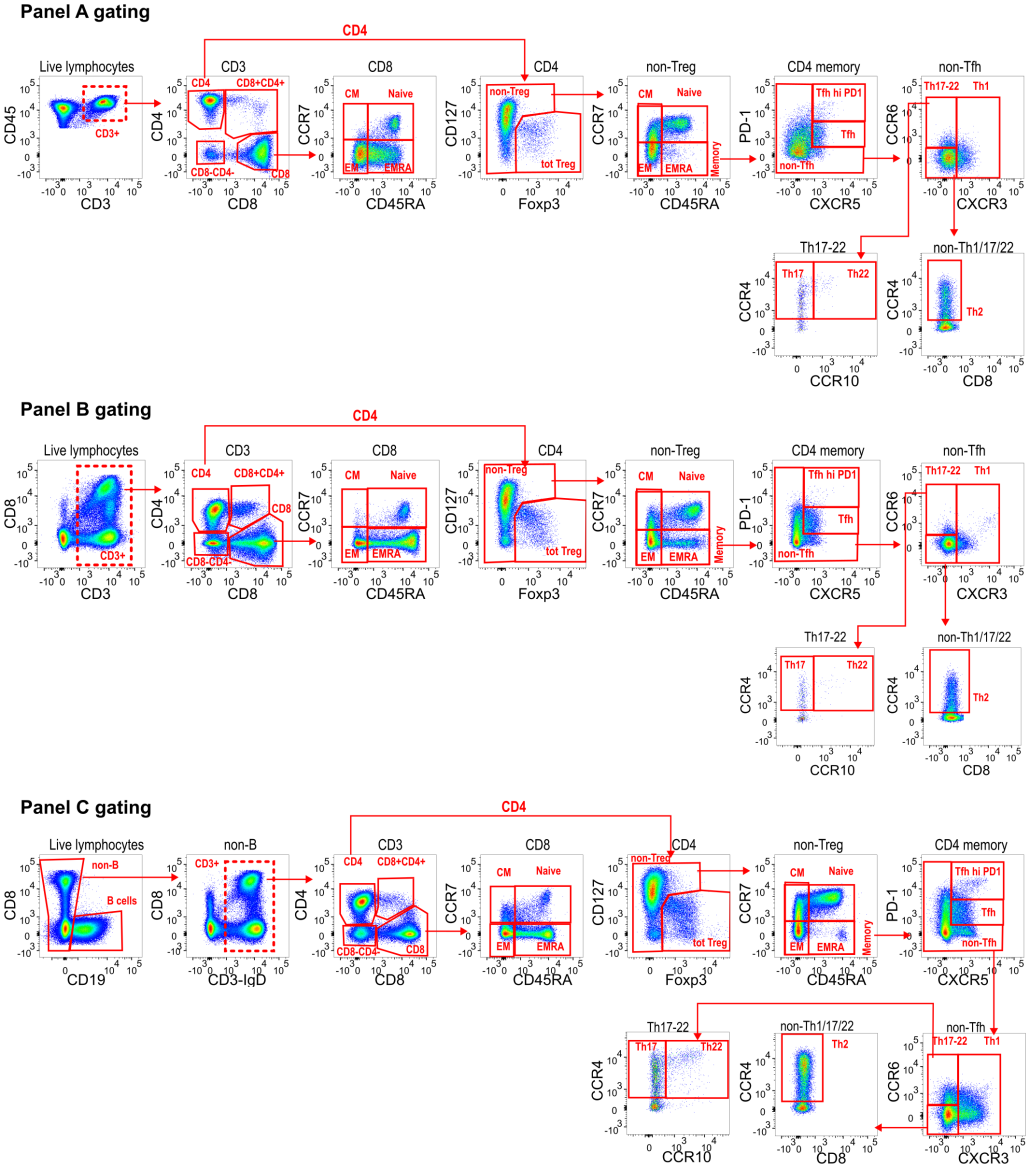
Panel-specific gating strategies created in FlowJo. For downstream DR analysis in FlowAtlas, we exported only live single T-cell events from each panel, indicated with a dashed line gate **(A)** Live CD3+ CD45+; **(B)** Live CD3+; **(C)** Live CD19- CD3+ events. Compensated parameters were exported, excluding CD45, CD19, Viability stain, FSC and SSC. Downstream gating for main population identification in FlowAtlas is shown. All channels have been biexponentially transformed. Note that FlowAtlas is compatible with biexponential transformation as implemented in FlowJo v10.8.1; other FlowJo transformations (e.g. logarithmic, ArcSinh) are not compatible with FlowAtlas.

STEP 5: Next, panel-specific gating hierarchy is created in FlowJo to define known populations of interest across all datasets (e.g. **Figure 2** for our example dataset). This is a user-supervised population-defining step and initial annotations typically represent high-level populations, such as naïve/memory B-cells, or CD4/CD8 memory T cells. Biexponential transformations should be applied to each channel in FlowJo, visually selecting the most appropriate width basis (co-factor) for each parameter in the dataset. FlowAtlas reads the biexponential transformations directly from FlowJo, enabling the user to set optimal population separation. This, in turn, has been shown to dictate the quality of dimensionality reduction and clustering (19). Matching populations, irrespective of panel, should be assigned the same annotation to enable cross-dataset pooling in FlowAtlas and analysis. Cells that fall outside of FlowJo-defined gates are auto-annotated as “Unlabelled” by FlowAtlas and can still be explored.

STEP 6: Finally, to facilitate data exploration, samples are grouped by conditions in FlowJo enabling FlowAtlas to filter and color-code embedded events. For our analysis, samples were grouped by donors and tissues (see Section 2.3).

STEP 7: The FlowJo workspace file is then imported into FlowAtlas (see instructions in Methods 4.6), which triggers dataset merging, DR, calculation of the embedding and launches an interactive browser interface (elements of FlowAtlas interface are shown in **Figure 1**, right panel).

**Figure 3:**
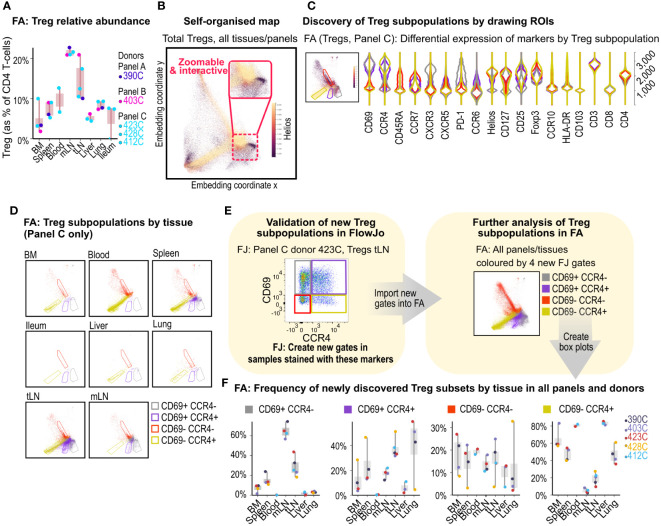
Treg subpopulation discovery in FlowAtlas. **(A)** Relative abundance of Tregs by donor and tissue calculated as % of total CD4+ T-cells. **(B)** Self-organized map embedding of Tregs from all tissues, all donors and all panels, colored by HELIOS expression. **(C)** Violin plots of 4 ROIs in the composite Treg embedding of all tissues stained with panel C; inset shows Tregs from all tissues stained with panel C, colored by CCR4 expression. **(D)** Treg ROI population distributions filtered by individual tissue. **(E)** Validation and creation of new Treg sub-gates for the four ROIs in FlowJo. Gates should be created in all samples that contain the markers of interest, regardless of panel, at equivalent levels in the gating tree hierarchy (e.g. the parent gate here is total Tregs). The new gates can then be opened and explored in FlowAtlas, as shown- Treg embedding re-colored by the newly annotated Treg populations. **(F)** Frequencies of the newly identified Treg subpopulations across tissues and donors. BM, bone marrow; mLN, mesenteric lymph nodes; tLN, thoracic lymph nodes; ROI, region of interest; FJ, FlowJo; FA, FlowAtlas.

The user interface displays the embedding map, which can be zoomed and panned to reveal fine cluster substructure. The FlowAtlas menu has four tabs: “Annotations”, “Expression”, “Frequency” and “Settings”. The “Annotations” tab enables cell filtering and re-coloring by sample groupings created in FlowJo, by cell population, or by heat-map of any marker expression. The filters can also be renamed or re-ordered here by dragging-and-dropping. The “Expression” tab has a polygon tool that enables drawing of multiple ROIs directly in the embedding to produce overlaid violin plots (**Figure 1**, right bottom inset) that reveal differences in marker expression thus enabling rapid identification of clusters with unique signatures. In the “Frequency” tab frequency box plots can be generated with a few clicks (e.g. **Figure 3A** and **Supplementary Figure S6** for our example dataset) showing frequencies of selected populations relative to their sum or any other population. Box plot marker colors and categories displayed on the x-axis are defined by filter selections in the “Annotations’’ tab. These features enable “on-the-fly”, intuitive exploration and analysis of complex datasets. All figures can be exported as publication-quality scalable vector graphics (SVG).

Once unique subpopulations and their signatures have been identified, they can be validated in FlowJo with targeted two-parameter plots and new population gates created to be read by FlowAtlas at rerun. This “iterative discovery loop” substantially simplifies and accelerates discovery.

**Figure 4:**
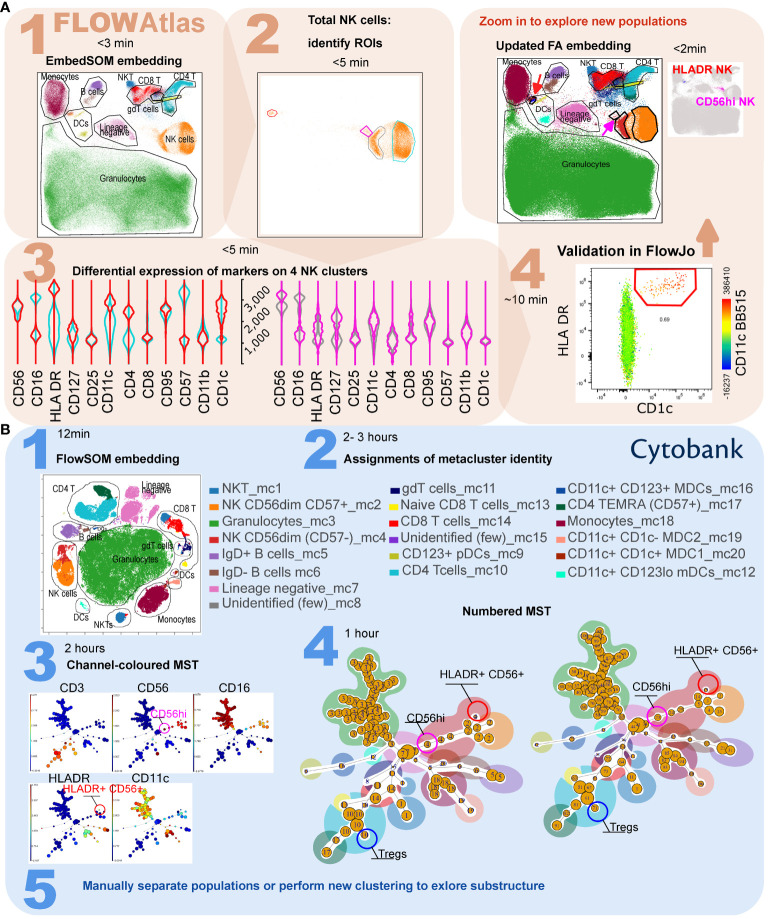
Comparison of workflow for the detection of rare cell subsets in FlowAtlas and Cytobank using a published spectral cytometry 23-colour dataset of whole human blood. **(A)** In FlowAtlas, embedding the data is quick. Basic populations are gated in FlowJo (Step 1A). Clusters in heterogeneous populations easily stand out on visual inspection, e.g. NK cells (Step 2A). A small cluster of NK cells (magenta ROI and violins) expresses HLA-DR, CD11c and CD1c (Step 3A). Validating its existence in FlowJo (Step 4A) is easy. The FlowAtlas embedding is re-opened with the new NK cell population. The larger NK subsets (CD56bright, CD57+ CD56dim and CD57- CD56dim) are zoomable. **(B)** Equivalent workflow in Cytobank: embedding is fast (step 1B). The user then annotates 20 metacluster populations by examining heatmaps and violin plots of marker expression (step 2B, process not shown). Rare populations, e.g. HLADR+ NK cells and CD56hi NK cells, may not have segregated. They can be discovered by examining the MST, colored by channel and cluster number (Step 3B and 4B), e.g. metacluster 4 contains cluster 15, expressing HLA-DR and CD56. To separate these events into a metacluster, the user should either re-run the analysis, or use Boolean commands to combine cluster numbers into a new population (Step 5B). Equivalent major cell populations are colored identically in the two embeddings and the minimum-spanning trees in Step 4B; ROI color in FlowAtlas matches the corresponding violin plots.

Hereafter, we demonstrate the capabilities of FlowAtlas using our novel conventional cytometry dataset of multi-donor multi-tissue derived immune cells or, where specified, other published datasets. Utilization of FlowAtlas for analysis of spectral and CyTOF data is shown in **Figure 4** and **Supplementary Figure S7** respectively.

### 2.3 Demonstrating the utility of FlowAtlas using example data

#### 2.3.1 Example cell population exploration

Our dataset consists of 32 files of tissue-derived immune cells obtained from 5 deceased transplant organ donors (**Supplementary Table S1**), stained with 3 different panels (**Supplementary Table S2**). The data were pre-processed in FlowJo as described in the “Recommended workflow” section to remove anomalous events, debris and aggregates; compensation was checked; and live, single T-cells were exported as new FCS files for downstream analysis. These files were imported into a new FlowJo workspace, and each channel was biexponentially transformed, basic populations were gated (**Figure 2**), and samples were grouped by donor ID and source tissue. Next, DR and clustering were performed in FlowAtlas. After generating relative abundance boxplots of the major lymphocyte populations in our dataset (**Supplementary Figure S6**), we elected to zoom into the CD4 regulatory T-cell (Treg) compartment, defined as CD3^+^CD4^+^CD127^-/lo^FOXP3^+^ cells, as an exemplar.

As a proportion of all CD4^+^ T-cells, Tregs were demonstrated to be enriched in lymph nodes, particularly mesenteric lymph nodes where they accounted for more than 20% of CD4 T-cells in all studied donors (**Figure 3A**).

The embedding of Tregs for Panel C donors, recolored by the expression of the transcription factor HELIOS (**Figure 3B**), revealed the presence of HELIOS^+^ and HELIOS^-^ subpopulations as expected (20, 21), with additional subcluster structures. Next, we filtered the embedding by panel C samples and used it to explore Treg subcluster characteristics further. We colored embedded events by tissue of origin and drew ROIs around four main subclusters seen in the embedding (**Figure 3C**). Auto-generated violin plots quickly allowed us to observe differences in expression of CD45RA, CCR7, CCR4 and CD69 between these subclusters, with the red ROI having a naive phenotype (CD45RA^+^CCR7^+^) and lacking CCR4 and CD69 expression, while yellow, grey and violet ROIs showed characteristics of memory subsets (CD45RA^-/lo^CCR7^-^) with and without CD69 and CCR4 expression. Filtering the embedding by tissue with the above ROIs superimposed (**Figure 3D**), revealed tissue-specific enrichment patterns; for example, CD69^+^ subsets were largely absent from blood, consistent with the role of CD69 in promoting tissue retention (22–24), whereas liver, lung, and thoracic lymph nodes contained a high proportion of Tregs expressing the chemokine receptor CCR4^+^ (with or without CD69 co-expression).

CCR4 has been implicated in T-cell trafficking to the lung (25), and in the infiltration of Tregs into tumors (26). Next, we validated the presence of these four Treg subsets in FlowJo (**Figure 3E**) and created new gates using CCR4 and CD69- now in all samples stained with these markers, irrespective of panel- for further exploration in FlowAtlas. Returning to FlowAtlas, we re-colored the Treg embedding by these newly annotated subsets and generated frequency box plots (**Figure 3F**), which further highlighted tissue-specific expression patterns.

FlowAtlas allowed us to obtain deep insights into the Treg population rapidly and intuitively. Therefore, we applied a similar analysis strategy to CD4^+^ Th1 and CD8^+^ memory cells, producing further data in a matter of minutes (**Supplementary Figures S4**, **S5**). This contrasts with analysis solely performed within FlowJo, where the computation of our full dataset embedding of 3.88 million events using tSNE would have been prohibitively slow (6 hours, see **Table 1** for comparison of performance) and assessing all possible combinations of markers using two-dimensional plots would have been a laborious process.

Thus, FlowAtlas offers two key advantages that considerably speed up data exploration: i) the embedding geometry is shared across all samples, even if they were stained with slightly different panels; ii) the eye is quickly drawn to patterns in the color or geometry of the 2D map that stand out- and the user can directly interact with these ROIs and assign their identities with relative ease, since the parent population is already known (set by the user in FlowJo).

#### 2.3.2 Detection of rare cell subsets using FlowAtlas

As explained above, current DR computational pipelines reduce computation time by down-sampling large datasets, which may not optimally reflect the distribution of the original data (27). Rare cell subsets may be missed by down-sampling and underfitting in existing unsupervised clustering approaches. Since FlowAtlas does not down-sample, it potentially circumvents this problem.

Accordingly, we next tested the ability of FlowAtlas to discover novel rare cell populations in the above-mentioned 23-parameter spectral cytometry dataset of whole human blood (12). As described, we performed the analysis in FlowAtlas and then replicated the example FlowSOM analysis demonstrated in Cytobank from curated experiment number 191382. The gating strategy for this dataset is shown in **Supplementary Figure S2**. Using FlowAtlas, we identified a subset of HLA-DR^+^ NK cells, comprising only 0.69% of total NK cells in under 30 min (**Figure 4**, steps 1A-4A). The same population was not resolved as a separate in Cytobank FlowSOM-on-viSNE analysis at the implemented settings (**Figure 4**, steps 1B and 2B). Furthermore, CD56^bright^ NK cells, which are well known to be phenotypically and functionally distinct (28), also did not segregate at these analysis settings.

In order to find the missing HLA-DR^+^ CD56^+^ subpopulation in Cytobank, it was necessary to review the 10 individual clusters comprising CD56^+^ events, which we colored by each parameter median fluorescence intensity (MFI) on the minimum spanning tree (MST, the tree-like graphical representation of the phenotypic similarities between cell populations). This was a time-consuming process. In FlowSOM analysis, related clusters of cells are organized into bigger groups called metaclusters. We noted that cluster 15 (a part of metacluster 4) was located away from the main metacluster 4 nodes and that it contained a small subset of HLA-DR^+^ CD56^+^ NK cells (**Figure 4**, step 3B and 4B). These may be the equivalent population to the cells discovered in FlowAtlas. We verified that the other 9 neighboring NK-cell clusters did not contain this population, by examining scatter plots of their key identifying markers (HLA-DR, CD11c) versus cluster number (not shown). Finally, we isolated the subpopulation manually based on its cluster number. This process took several hours and was informed by our prior identification of this population in FlowAtlas.

Resolution of other rare populations would potentially require each of the 100 clusters to be individually examined, as above. Once discovered, a rare subpopulation would either need to be manually separated (by combining clusters with Boolean commands), or the analysis needs to be repeated from the beginning with different settings or starting with smaller more homogenous cell population (e.g. only NK cells). By contrast, FlowAtlas allows the user to simply zoom in on the existing embedding to study the substructure of clusters without needing to re-embed the data.

#### 2.3.3 FlowAtlas can integrate multiple flow cytometry panels, but protocol-driven experiment harmonization remains critical

##### 2.3.3.1 Integration of datasets stained with different panels

During this project, our panel design evolved, so that our final tissue-derived immune cell dataset consisted of 3 different panels. Most existing computational tools require the files to be combined (concatenated) prior to analysis, which is impossible when different markers have been assigned to the same fluorochrome (i.e. cytometer detector channel). This would typically cause researchers to exclude precious data that they cannot integrate. Therefore, it was essential that we engineered FlowAtlas with the capability to handle datasets stained with slightly discrepant panels. We will now discuss how this was achieved, as well as the limitations within which this feature operates.

FlowAtlas enables data re-use and concomitant analysis of datasets acquired with non-identical antibody panels by imputing missing values using random sampling with replacement before DR. Algorithmic bias (i.e. synthetic data that result purely from the imputation and are not physically present in the biological sample) is prevented by excluding imputed values from the embedding visualization or any downstream analyses.

To demonstrate the capability to merge panels, we acquired 2 healthy control blood samples and stained them with the 3 panels previously used in our main tissue-derived dataset. The use of the same two donors with all 3 panels eliminated any biological variation, enabling us to isolate the effect of panel differences within the healthy control group. We integrated the 6 new FCS files (1.28 million live single T-cell events) into the existing embedding of tissue-derived immune cells.

**Figure 5:**
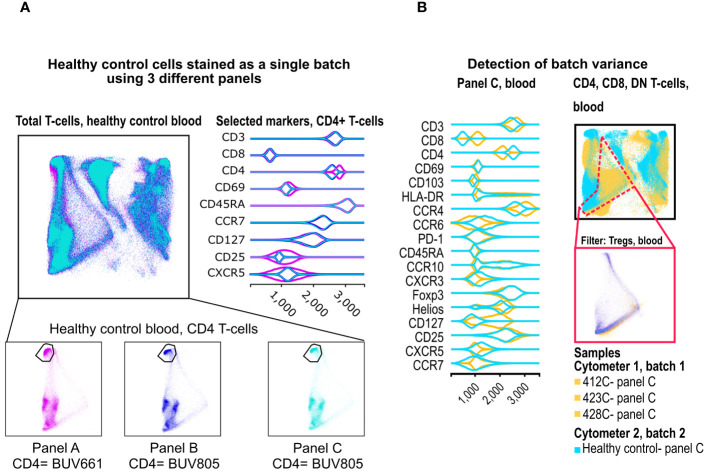
Merging of panels and detection of batch variance. **(A)** Two healthy control donors were stained with our 3 panels as one batch, and data were processed in FlowAtlas as recommended. Events are colored by panel and show minimum differences in population geometry, driven by our choice of CD4 fluorochrome (BUV661 on panel A, BUV805 on panels B and C). The BUV661 fluorochrome spreads signal into CD25-APC and CXCR5-APCR700. This is visible in the violin plots of panel A, where the CD25-negative population is wider (i.e. there is less separation between positive and negative due to the design of this panel). The three panels in this example integrate well without computational batch correction because of protocol-based steps taken to avoid major technical variability **(B)** Blood samples stained with panel C are shown as embedding and violin plots (yellow= deceased organ donor blood, processed ex vivo, “batch 1”; cyan= healthy control blood, processed after cryopreservation, “batch 2”). FlowAtlas has successfully merged the panels, but the resulting topography of the two batches is different, reflecting a mixture of biological differences and technical differences in batch handling.

We filtered the embedded data by “healthy control” so that only the healthy samples are displayed. Then, we colored the embedding by panel and inspected differences in cluster position, geometry and marker mean fluorescence intensity (MFI). We noted: i) very slight variation in cluster position that results from the use of different fluorochromes for CD4 (see **Supplementary Tables S2**, **S3**); ii) some differences in violin plots, particularly wider negative populations (due to differences in data spread in the two panels, a phenomenon explained in **Figure 5** legend). Nevertheless, the overall embedding geometry was highly conserved across the three panels (**Figure 5A**).

We also tested whether extremely different panels can be combined (for details, see **Supplementary Figure S7**). Panels with very few shared markers and/or fluorochromes could be processed by FlowAtlas. However, equivalent cell populations failed to co-localize adequately, due to a lack of common landmarks between the datasets (**Supplementary Figure S8**).

In summary, FlowAtlas is relatively robust at handling samples with mildly to moderately different panels, but optimum co-localization of equivalent populations requires relatively conservative panel discrepancies.

##### 2.3.3.2 Integration of datasets with major technical batch effects

As mentioned in Section 2.2, FlowAtlas was designed without a built-in batch correction step, and users have to account for this experimentally or computationally. Our tissue-derived dataset was successfully integrated with minimal batch effects because of careful staining protocol harmonization and day-to-day cytometer calibration.

To emphasize this point, we stained a set of healthy control blood samples with the same three panels used in our deceased organ donor tissue dataset. However, unlike the tissue-derived dataset, healthy PBMCs had been cryopreserved and acquired on a cytometer with a different optical configuration (See Methods and **Supplementary Table S4**). These experimental discrepancies were expected to produce an extreme example of batch effects.

To illustrate the batch differences, we embedded the healthy control blood, and deceased donor tissue datasets on the same DR map. All data preparation steps and gating strategy were otherwise identical. In FlowAtlas, we displayed only blood-derived cells stained with panel C. We colored the samples by group (healthy controls vs deceased organ donors, **Figure 5B**). There were significant qualitative differences in the embedding geometry for these two sets of samples. The resulting violin plots showed differences in several chemokine receptors, CD127, CD4 and CD8. Although biological differences between healthy and deceased donor blood may contribute to this observation, the magnitude of the differences strongly suggested they were driven by batch effects.

In summary, FlowAtlas does not perform batch correction, and, though it can still compute a map for the combined data, the batch differences render the resulting map geometry of the combined data difficult or impossible to interpret. Therefore, it is essential that users experimentally control for, or computationally correct, batch effects in their dataset before using FlowAtlas (a computational approach is demonstrated in **Supplementary Figure S9**).

## 3 Discussion

FlowAtlas is a novel open-source data exploration tool, which combines the computational power of the GigaSOM library and Julia programming language with the widely used software FlowJo, expanding its capabilities in a completely graphical, fast, user-friendly interface. This approach removes all entry barriers imposed by command-line analysis pipelines that currently hold many users back from taking advantage of powerful computational tools. FlowAtlas brings a new iterative analysis concept to biomedical scientists by linking the familiar FlowJo workflow with a high-performance machine learning framework. FlowAtlas allows rapid computation of millions of high-dimensional events without the need for down-sampling. The highly interactive embedding enables zooming and intuitive exploration of population substructure, considerably speeding up population discovery. Missing-data handling methods enable concomitant analysis of datasets with non-identical panel designs or markers. Importantly, FlowAtlas does not incorporate batch correction, and, to prevent algorithmic bias, does not display imputed values in the embedding. Here, we briefly discuss the rationale behind our design decision.

As emphasized throughout this work, data preparation is crucial to successful analysis in FlowAtlas and this includes: i) removal of irrelevant events such as debris, aggregates and dead cells; ii) optimal compensation for each file; iii) correction of technical (non-biological) variation between samples. Therefore, it may appear surprising that we designed FlowAtlas without an integrated batch correction step. This was a deliberate choice that enables users to select the most appropriate method for their specific experimental context.

Best practice for minimization of batch-effects currently relies on inter-laboratory protocol harmonization through the use of standardized antibody cocktails, identical staining procedures, calibration of cytometers using fluorescence standards or Application Settings (29). Protocol-based approaches, such as those we used to acquire our tissue-derived immune cell dataset, would likely best suit biologists- the primary target user demographic of FlowAtlas- as they circumvent the need for coding.

Alternatively, batch correction is possible using computational methods, but this can often be more challenging. Tools such as swiftReg in R (30), and CytoNorm (18) and CyCombine (17), which are both available as plugins in FlowJo, are examples of batch correction algorithms. CytoNorm requires biological “anchor” controls stained with each batch of samples to correct the fluorescence intensity of markers in each sample. Due to concerns that this may eliminate some biologically relevant fluorescence differences, this pipeline is suitable for analyzing population frequency (not fluorescence intensity) as the main variable of interest.

In the absence of internal anchor controls, the currently available computational methods of batch correction need considerable command-line competence. For example, GaussNorm (in R) aligns cellular landmarks (positive and negative population peaks) across samples (31). Powerful batch correction tools rooted in single-cell genomics packages are now finding application in flow and mass cytometry, e.g. Seurat in R (32) and Pytometry in Python (33). The stringency of batch effect removal versus biological effect preservation varies widely between these methods (34), so the optimum batch correction pipeline may vary between datasets. For this reason, we chose not to integrate any particular computational batch correction pipeline with FlowAtlas, allowing users to choose if they require this step, and how best to approach it during experiment design and data pre-processing.

With respect to panel merging, the missing-data handling methods in FlowAtlas ensure it is relatively robust to moderate panel differences, enabling dataset integration in selected circumstances. We substituted some markers in our panels and demonstrated that FlowAtlas can preserve the embedding geometry under the tested conditions. Nevertheless, panels with little overlap in markers or fluorochromes are unlikely to integrate successfully. Where multiple markers differ, users are advised to test the effectiveness of panel integration by staining a single donor sample with their panels of interest and assessing the resulting embedding geometry. Tools have been developed, which aim to combine panels through marker imputation, e.g. CyCombine (17), CytoBackBone (35), CyTOFMerge (36) and Infinicyt (Cytognos, BD). Nevertheless, we chose not to display imputed values in the FlowAtlas embedding to protect against algorithmic bias. A critical assessment of these methods has recently reported relatively poor approximation of known expression values (37), justifying our decision.

In conclusion, FlowAtlas is a novel data exploration tool, which leverages advanced machine learning methods, rapid computational speed, and a near-complete lack of a user learning curve before data exploration can commence. The highly interactive and intuitive workflow eliminates the need for command-line coding and brings high-dimensional data exploration and population discovery to the non-bioinformatician biologist.

## 4 Materials and methods

### 4.1 Ethical statement

All work was completed under ethically approved studies. Healthy human PBMCs were isolated from volunteers having given informed consent under CAMSAFE (REC- 11/33/0007). All deceased organ donor tissue samples were collected via the Cambridge Biorepository for Translational Medicine under Research Ethics Committee approval 15/EE/0152. In addition, two donor-matched blood samples were collected prior to withdrawal of life support, under Ethics Committee approval 97/290.

### 4.2 Tissue acquisition and dissociation, and preparation of healthy control PBMCs

Tissue was obtained from five deceased organ donors following circulatory death. Donor metadata is given in **Supplementary Table S1**, and a graphical summary of all samples and data sources is in **Supplementary Figure S10**. Briefly, following cessation of circulation, human donor organs were perfused *in situ* with cold organ preservation solution and cooled with topical application of ice. Samples for the study were obtained within 60 minutes of cessation of circulation and placed in University of Wisconsin organ preservation solution for transport at 4°C to the laboratory. Lung and liver samples were obtained from the left lower lobe of the lung and the right lobe of the liver. In addition, two donor-matched blood samples were collected prior to withdrawal of life support (under REC approval 97/290). To minimize the possibility of processing-dependent differences in cell surface marker expression, all samples, including blood, were processed using enzymatic digestion protocol. Briefly, solid tissues were weighed, transferred into 10cm tissue culture dishes, and cut into small pieces. Up to 5g of tissue was then transferred into a GentleMACS C tube (Miltenyi Biotec) prefilled with 5mL of dissociation media composed of X-VIVO15 with 0.13U/mL Liberase TL (Roche), 10U/mL Benzonase nuclease (Millipore/Merck), 2% (v/v) heat-inactivated fetal bovine serum (FBS, Gibco), penicillin (100 U/ml, Sigma-Aldrich), streptomycin (0.1 mg/ml, Sigma-Aldrich), and 10mM HEPES (Sigma Aldrich). The samples were then homogenised using a GentleMACS Octo dissociator (Miltenyi Biotec) running a protocol that provided gradual ramping up of homogenization speed and two 15-minute heating/mixing steps at 37°C. Digested tissue was passed through a 70μm MACS Smartstrainer (Miltenyi Biotec) and the flow-through was first washed with X-VIVO15 supplemented with 2 mM EDTA and then with PBS. Mononuclear cells were enriched by Ficoll-Paque (GE Healthcare) density centrifugation according to the manufacturer’s instructions. Following density centrifugation, mononuclear layer was collected, washed once with PBS and the cell pellet was resuspended in FACS buffer (PBS, 2.5% FBS). Bone marrow aspirates and peripheral blood samples were first subjected to Ficoll-Paque density centrifugation, according to manufacturer’s instructions, the mononuclear layer was then collected, washed with PBS and cells were treated with the same dissociation media as solid tissues for 30 min at 37°C prior to washing and resuspension in FACS buffer.

Healthy control PBMCs were prepared by Ficoll-gradient centrifugation and cryopreserved in cell freezing medium (Sigma) containing 10% DMSO for future use.

### 4.3 Flow cytometry of tissue-derived mononuclear cells

Depending on the cell yield, up to 1x10^6^ mononuclear cells/tissue were stained with antibodies shown in **Supplementary Table S2**. Not all donors were stained with the same panel. To expand the total number of markers, sentinel panel design was implemented where CD3 and IgD were detected with antibodies conjugated to BUV395 and FOXP3 and IgM were detected with antibodies conjugated to PE in some donors. Refer to **Supplementary Table S2** for details. Single cell suspensions were washed once in PBS, transferred into 96 v-bottom plate and stained with Zombie UV viability dye for 30 min at 4°C followed by a wash with FACS buffer. Cell pellets were resuspended in 50μl FACS buffer with Human FcR block (BD Biosciences) and incubated for 10 min at 4°C. Next, cells were pelleted, excess buffer removed and 100μl of antibody master mix composed of cell-surface antibody cocktail (see **Supplementary Table S3**), BV buffer (BD) and True-Stain Monocyte Blocker (Biolegend) and incubated for 1h at 4°C. Following incubation, cells were washed three times in PBS and prepared for intracellular staining using transcription factor fixation/permeabilization kit (eBioscience) according to the manufacturer’s instructions. Following intracellular staining, cells were resuspended in PBS and analyzed on BD FACSymphony A3 cell analyzer within 10 hours.

### 4.4 Flow cytometry of healthy PBMCs

In contrast to tissue-derived samples, which were processed *ex vivo*, healthy PBMC samples were thawed in X-VIVO15/10% FCS at room temperature and stained according to the procedure above. Analysis was performed on a BD FACSymphony A5 cell analyzer within 10 hours. The optical configuration of the two cytometers used in this study is shown in **Supplementary Table S4**. The cytometers were not cross-calibrated for comparable measurement of MFI, but each underwent individual CS&T bead quality control before sample acquisition.

### 4.5 FlowAtlas code availability

The code for FlowAtlas is open-source and is available at our GitHub repository: https://github.com/gszep/FlowAtlas.jl.git

### 4.6 Installation and loading of FlowAtlas

FlowAtlas is compatible with FlowJo version 10.8.1.

FlowAtlas requires Julia language, which is easily installed on any operating system by downloading an installer available here: https://julialang.org/downloads and following the on-screen instructions. Tick the option to add Julia to PATH environment when prompted.

Once Julia is installed, FlowAtlas can be installed and run in three lines of code as follows:

1. Windows: open Run (Windows Key + R), type cmd and hit enter. MacOS: open command prompt (Cmd Key + Space), type terminal and hit enter. This will launch Windows/MacOS command prompt.
2. In the prompt type Julia and hit enter. This will launch the Julia environment.
3. Type] and the prompt will change to display that package manager is now active.
4. Type add FlowAtlas and hit enter. This will download and install FlowAtlas. Once installation is complete, you can close the command prompt window.

To start using FlowAtlas, navigate to the folder containing your pre-processed FCS files (make sure that the FlowJo workspace file is there as well) and launch command prompt as follows: in Windows by typing cmd in the File Explorer address bar (where file path is usually displayed) and hitting enter or in MacOS launch terminal and navigate to the folder by typing cd followed by the folder path. In the prompt, type Julia and hit enter to start it, then type using FlowAtlas and hit enter. Once FlowAtlas is loaded, type FlowAtlas.run(“workspace.wsp”; files=“*/*.fcs”) where workspace.wsp is the name of your FlowJo analysis file with.wsp extension. Adding new files into the workspace after initial analysis will force a recalculation of the embedding.

Embedding is performed only once when the workspace file is first imported and is stored in a cache file with a “.som” extension, allowing users to return to their analysis quickly. The embedding can also be re-calculated to change cluster geometry (by removing the.som file from the working folder and initiating the programme again). Sharing the “.som” file together with the FlowJo workspace and FCS files enables collaboration, allowing colleagues to work on the same embedding map.

A short video demonstrating the use of FlowAtlas can be watched here: https://www.youtube.com/watch?v=FeYrFKgP91s.

### 4.7 Processing large datasets with FlowAtlas

Computation time in FlowAtlas increases as a function of total event number in the entire dataset (the number of events per file is irrelevant), and to a lesser extent, data complexity. Very large datasets can be processed given sufficient RAM. As an approximate guide, on a laptop configured with 64-bit Windows OS, 64GB RAM, 14-core i7-13700H processor, we noted the following processing times: 500,000 events (32 parameters)= 4 min; 9 million events (10 parameters)= 23 min; 17.3 million events (32 parameters)= 25 minutes. 46 million events (25 parameters) = 113 minutes. A video of real-time exploration of the largest dataset is available here: https://youtu.be/0soJw8PT2bU?feature=shared.

## Data availability statement

The datasets presented in this study can be found in online repositories. The names of the repository/repositories and accession number(s) can be found below: FlowRepository http://flowrepository.org/id/FR-FCM-Z74D.

## Ethics statement

The studies involving humans were approved by East of England Research Ethics Committee. The studies were conducted in accordance with the local legislation and institutional requirements. The participants provided their written informed consent to participate in this study.

## Author contributions

VC: Conceptualization, Data curation, Formal analysis, Investigation, Methodology, Validation, Visualization, Writing – original draft, Writing – review & editing. GS: Conceptualization, Methodology, Software, Writing – review & editing. ZG: Conceptualization, Data curation, Formal analysis, Investigation, Validation, Visualization, Writing – original draft, Writing – review & editing, Methodology. SH: Data curation, Investigation, Methodology, Writing – review & editing. LJ: Data curation, Investigation, Methodology, Writing – review & editing. DR: Conceptualization, Data curation, Investigation, Methodology, Writing – review & editing. OS: Data curation, Writing – review & editing, Resources. EN: Resources, Writing – review & editing, Investigation. HM: Resources, Writing – review & editing, Investigation. DM: Resources, Supervision, Writing – review & editing. FF: Methodology, Software, Writing – review & editing. KM: Resources, Writing – review & editing. KS-P: Resources, Supervision, Writing – review & editing. JJ: Conceptualization, Funding acquisition, Project administration, Resources, Supervision, Writing – review & editing.

## References

[1] der Maaten L Hinton G. Visualizing Data using t-SNE. JMLR. (2008) 9:2579–605.

[2] Becht E McInnes L Healy J Dutertre C-A Kwok IWH Ng LG. Dimensionality reduction for visualizing single-cell data using UMAP. Nat Biotechnol. (2019) 37:38–44. doi: 10.1038/nbt.4314 30531897

[3] Kohonen T. Essentials of the self-organizing map. Neural Networks. (2013) 37:52–65. doi: 10.1016/j.neunet.2012.09.018 23067803

[4] Bezanson J Edelman A Karpinski S Shah VB. Julia: A fresh approach to numerical computing. SIAM Rev. (2017) 59:65–98. doi: 10.1137/141000671

[5] Roesch E Greener JG MacLean AL Nassar H Rackauckas C Holy TE. Julia for biologists. Nat Methods. (2023) 20:655–64. doi: 10.1038/s41592-023-01832-z PMC10216852 37024649

[6] Kratochvíl M Hunewald O Heirendt L Verissimo V Vondrášek J Satagopam VP. GigaSOM.jl: High-performance clustering and visualization of huge cytometry datasets. GigaScience. (2020) 9:giaa127. doi: 10.1093/gigascience/giaa127 33205814 PMC7672468

[7] Kratochvíl M Koladiya A Vondrášek J. Generalized EmbedSOM on quadtree-structured self-organizing maps. F1000Res. (2020) 8:2120. doi: 10.12688/f1000research.21642.2 PMC7255855 32518625

[8] Van Gassen S Callebaut B Van Helden MJ Lambrecht BN Demeester P Dhaene T. FlowSOM: Using self-organizing maps for visualization and interpretation of cytometry data. Cytomet Part A. (2015) 87:636–45. doi: 10.1002/cyto.a.22625 25573116

[9] Chen T Kotecha N. “Cytobank: providing an analytics platform for community cytometry data analysis and collaboration.,”. In: High-dimensional single cell analysis. Springer, Berlin, Heidelberg. (2014). doi: 10.1007/82_2014_364 24590675

[10] . OpenLayers. Available online at: https://openlayers.org/ (Accessed November 19, 2023).

[11] Bostock M Ogievetsky V Heer J. D^3^ data-driven documents. IEEE Trans Visual Comput Graphics. (2011) 17:2301–9. doi: 10.1109/TVCG.2011.185 22034350

[12] Cytek Aurora - 23 color immunophenotyping (FlowSOM demo) - Experiment summary - Cytobank . Cytobank. Available online at: https://premium.cytobank.org/cytobank/experiments/191379 (Accessed November 19, 2023).

[13] Monaco G Chen H Poidinger M Chen J De Magalhães JP Larbi A. flowAI: automatic and interactive anomaly discerning tools for flow cytometry data. Bioinformatics. (2016) 32:2473–80. doi: 10.1093/bioinformatics/btw191 27153628

[14] Meskas J Yokosawa D Wang S Segat GC Brinkman RR. flowCut: An R package for automated removal of outlier events and flagging of files based on time versus fluorescence analysis. Cytomet Part A. (2023) 103:71–81. doi: 10.1002/cyto.a.24670 PMC9823164 35796000

[15] Fletez-Brant K Špidlen J Brinkman RR Roederer M Chattopadhyay PK. flowClean: Automated identification and removal of fluorescence anomalies in flow cytometry data. Cytomet Part A. (2016) 89:461–71. doi: 10.1002/cyto.a.22837 PMC5522377 26990501

[16] Emmaneel A Quintelier K Sichien D Rybakowska P Marañón C Alarcón-Riquelme ME. PeacoQC: Peak-based selection of high quality cytometry data. Cytomet A. (2022) 101:325–38. doi: 10.1002/cyto.a.24501 PMC9293479 34549881

[17] Pedersen CB Dam SH Barnkob MB Leipold MD Purroy N Rassenti LZ. cyCombine allows for robust integration of single-cell cytometry datasets within and across technologies. Nat Commun. (2022) 13:1698. doi: 10.1038/s41467-022-29383-5 35361793 PMC8971492

[18] Van Gassen S Gaudilliere B Angst MS Saeys Y Aghaeepour N. CytoNorm: A normalization algorithm for cytometry data. Cytomet Part A. (2020) 97:268–78. doi: 10.1002/cyto.a.23904 PMC7078957 31633883

[19] Finak G Perez J-M Weng A Gottardo R. Optimizing transformations for automated, high throughput analysis of flow cytometry data. BMC Bioinf. (2010) 11:546. doi: 10.1186/1471-2105-11-546 PMC3243046 21050468

[20] Himmel ME MacDonald KG Garcia RV Steiner TS Levings MK. Helios+ and helios– cells coexist within the natural FOXP3+ T regulatory cell subset in humans. J Immunol. (2013) 190:2001–8. doi: 10.4049/jimmunol.1201379 23359504

[21] Thornton A. Helios+ and Helios– Treg subpopulations are phenotypically and functionally distinct and express dissimilar TCR repertoires. Eur J Immunol. (2019) 49:398–412. doi: 10.1002/eji.201847935 30620397 PMC6402968

[22] Shiow LR Rosen DB Brdičková N Xu Y An J Lanier LL. CD69 acts downstream of interferon-α/β to inhibit S1P1 and lymphocyte egress from lymphoid organs. Nature. (2006) 440:540–4. doi: 10.1038/nature04606 16525420

[23] Kumar BV Ma W Miron M Granot T Guyer RS Carpenter DJ. Human tissue-resident memory T cells are defined by core transcriptional and functional signatures in lymphoid and mucosal sites. Cell Rep. (2017) 20:2921–34. doi: 10.1016/j.celrep.2017.08.078 PMC5646692 28930685

[24] Sathaliyawala T Kubota M Yudanin N Turner D Camp P Thome JJC. Distribution and compartmentalization of human circulating and tissue-resident memory T cell subsets. Immunity. (2013) 38:187–97. doi: 10.1016/j.immuni.2012.09.020 PMC3557604 23260195

[25] Mikhak Z Strassner JP Luster AD. Lung dendritic cells imprint T cell lung homing and promote lung immunity through the chemokine receptor CCR4. J Exp Med. (2013) 210:1855–69. doi: 10.1084/jem.20130091 PMC3754856 23960189

[26] Bromley SK Mempel TR Luster AD. Orchestrating the orchestrators: chemokines in control of T cell traffic. Nat Immunol. (2008) 9:970–80. doi: 10.1038/ni.f.213 18711434

[27] Lötsch J Malkusch S Ultsch A. Optimal distribution-preserving downsampling of large biomedical data sets (opdisDownsampling). PloS One. (2021) 16:e0255838. doi: 10.1371/journal.pone.0255838 34352006 PMC8341664

[28] Angelo LS Banerjee PP Monaco-Shawver L Rosen JB Makedonas G Forbes LR. Practical NK cell phenotyping and variability in healthy adults. Immunol Res. (2015) 62:341–56. doi: 10.1007/s12026-015-8664-y PMC4470870 26013798

[29] Kalina T Flores-Montero J van der Velden VHJ Martin-Ayuso M Böttcher S Ritgen M. EuroFlow standardization of flow cytometer instrument settings and immunophenotyping protocols. Leukemia. (2012) 26:1986–2010. doi: 10.1038/leu.2012.122 22948490 PMC3437409

[30] Rebhahn JA Quataert SA Sharma G Mosmann TR. SwiftReg cluster registration automatically reduces flow cytometry data variability including batch effects. Commun Biol. (2020) 3:1–14. doi: 10.1038/s42003-020-0938-9 32382076 PMC7205614

[31] Hahne F Khodabakhshi AH Bashashati A Wong C-J Gascoyne RD Weng AP. Per-channel basis normalization methods for flow cytometry data. Cytomet A. (2010) 77:121–31. doi: 10.1002/cyto.a.20823 PMC3648208 19899135

[32] Tools for single cell genomics . Satija Lab. Available online at: https://satijalab.org/seurat/ (Accessed November 19, 2023).

[33] Büttner M Hempel F Ryborz T Theis FJ Schultze JL. Pytometry: Flow and mass cytometry analytics in Python. bioRxiv (2022) 2022. doi: 10.1101/2022.10.10.511546

[34] Luecken MD Büttner M Chaichoompu K Danese A Interlandi M Mueller MF. Benchmarking atlas-level data integration in single-cell genomics. Nat Methods. (2022) 19:41–50. doi: 10.1038/s41592-021-01336-8 34949812 PMC8748196

[35] Leite Pereira A Lambotte O Le Grand R Cosma A Tchitchek N. CytoBackBone: an algorithm for merging of phenotypic information from different cytometric profiles. Bioinformatics. (2019) 35:4187–9. doi: 10.1093/bioinformatics/btz212 PMC6792066 30903138

[36] Abdelaal T Höllt T Van Unen V Lelieveldt BPF Koning F Reinders MJT. CyTOFmerge: integrating mass cytometry data across multiple panels. Bioinformatics. (2019) 35:4063–71. doi: 10.1093/bioinformatics/btz180 PMC6792069 30874801

[37] Mocking TR Duetz C van Kuijk BJ Westers TM Cloos J Bachas C. Merging and imputation of flow cytometry data: a critical assessment. Cytomet Part A. (2023) 103:818–29. doi: 10.1002/cyto.a.24774 37338802

