# Supplementary material for: FlowAtlas: an interactive tool for high-dimensional immunophenotyping analysis bridging FlowJo with computational tools in Julia
Source: Front Immunol. 2024 Jul 17;15:1425488. doi: 10.3389/fimmu.2024.1425488 (PMC11288863; doi:10.3389/fimmu.2024.1425488)

Supplementary Material

FlowAtlas: an interactive tool bridging FlowJo with computational tools in Julia

Valerie Coppard[1,†], Grisha Szep[2,†], Zoya Georgieva[1,†,*], Sarah K. Howlett[1] , Lorna B. Jarvis[1], Daniel B. Rainbow[1], Ondrej Suchanek[3], Edward J. Needham[1], Hani S. Mousa[1], David K. Menon[4], Felix Feyertag[5], Krishnaa T. Mahbubani[6,7], Kourosh Saeb-Parsy[6,7], and Joanne L. Jones[1,*]

† **These authors share first authorship:** VC, GS and ZG.

*** Correspondence:**

Corresponding Authors: zg248@medschl.cam.ac.uk , jls53@medschl.cam.ac.uk

1 Supplementary Tables

S1 Table: Donor characteristics

ID	Sex	Age	Cause of death	Multi trauma	Days in Hospital	BMI	CMV EBV TOXO	Smoker	Alcohol (u/day)	Antibiotic within 2 weeks of death	Steroid
390C	F	65-70	ICH	✓	2	30-35	+/+/-	?	<1	✗	✗
403C	M	50-55	ICH	✓	8	30-35	+/+/-	✓	<1	Co,T	✗
423C	M	60-65	ICH	✗	2	20-25	-/+/-	✓	>9	G,F	D
412C	M	70-75	ICH	✗	5	26-30	-/+/+	✓	<2	A*, F, G, C, Co	P†
428C	F	55-60	ICH	✗	3	20-25	-/+/-	✓	>9	Co	✗

F= Female; M= Male; ICH= intracranial haemorrhage; CMV= cytomegalovirus; EBV= Ebstein-Barr virus; TOXO= Toxoplasmosis; Co= co-amoxiclav; A= amoxicillin; T= Tazocin; F= Flucloxacillin; G= Gentamicin; D= Dexamethasone; C= Clarithromycin; ✓= yes;✗= no; ?= not known; P= Prednisolone; * pre-admission; † pre-treatment

S2 Table: Immunophenotyping panels used in this study

		Fluorochromes		
	Panels	A	B	C
Specificity	Donor(s)	390C	403C	412C, 423C, 428C
CD45		BUV805	-	-
CD19		-	-	BUV570
IgM		-	-	PE
IgD		-	-	BUV395
CD4		**BUV661**	**BUV805**	**BUV805**
CD3		BUV395	BUV395	BUV395
CD8		BUV563	BUV563	BUV563
CD69		BUV737	BUV737	BUV737
CD103		BV421	BV421	BV421
HLA-DR		BV510	BV510	BV510
CD127		PE-Cy7	PE-Cy7	PE-Cy7
CCR4		BV605	BV605	BV605
CCR6		BV650	BV650	BV650
PD-1		BV711	BV711	BV711
CD45RA		BV786	BV786	BV786
CCR10		BB515	BB515	BB515
CXCR3		BB700	BB700	BB700
CXCR5		APCR-700	APCR-700	APCR-700
CCR7		APC-Fire750	APC-Fire750	APC-Fire750
CD25		APC	APC	APC
FOXP3		PE	PE	PE
HELIOS		PE-Dazzle	PE-Dazzle	PE-Dazzle
Live/Dead		Zombie UV	Zombie UV	Zombie UV

Different fluorochromes were used for anti-CD4 in each panel (in bold). Not all antibodies were present in all panels. FOXP3 and IgM were both on PE in panel C.

S3 Table: Antibodies used in this study

Specificity	Fluorochrome	Clone	Source
CD3	BUV395	SK7	BD
IgD	BUV395	IA6-2	BD
CD8	BUV563	RPA-T8	BD
CD69	BUV737	FN50	BD
CD4	BUV661	SK3	BD
CD4	BUV805	SK3	BD
CD45	BUV805	HI30	BD
CD103	BV421	Ber-ACT8	BD
HLA-DR	BV510	G46-6	BD
CD19	BV570	HIB19	Biolegend
CCR4	BV605	L291H4	Biolegend
CCR6	BV650	11A9	BD
PD-1	BV711	EH12.1	BD
CD45RA	BV786	HI100	BD
CCR10	BB515	1B5	BD
CXCR3	BB700	1C6/CXCR3	BD
FOXP3	PE	259D/C7	BD
FOXP3	PE	PCH101	eBioscience
IgM	PE	G20-127	BD
HELIOS	PE-Dazzle594	22F6	Biolegend
CD127	PECy7	HIL-7R-M21	BD
CD25	APC	2A3	BD
CD25	APC	MA251	BD
CXCR5	APC-R700	RF8B2	BD
CCR7	APC-Fire750	G043H7	Biolegend
Viability	Live/Dead UV	-	Biolegend

S4 Table: Optical configuration of the cytometers used in this study

Laser	FACS Symphony A3	FACS Symphony A5	Utilised fluorochrome
Acquired samples	Tissue-derived immune cells	Healthy control PBMCs	
	Bandpass filter	Bandpass filter	
355nm	379 / 28	379 / 28	BUV395
		450 / 50	-
	515 / 30	515 / 30	Zombie UV
	560 / 40	580 / 20	BUV563
		610 / 20	-
	670 / 30	670 / 20	BUV661
	740 / 35	735 / 30	BUV737
	820 / 60	770 / 40	BUV805
405nm	450 / 50	431 / 28	BV421
	515 / 20	525 / 50	BV510
	585 / 15	585 / 15	BV570
	605 / 40	605 / 40	BV605
	670 / 30	677 / 20	BV650
	710 / 40	710 / 50	BV711
	741 / 40	750 / 30	
	780 / 60	770 / 40	BV786
488nm	488/10	488 / 10	
	525 / 50	530 / 30	BB515
	610 / 20	610 / 20	-
	685 / 35	670 / 30	-
	715 / 30	710 / 50	BB700
		750 / 30	-
	780 / 60	770 / 40	-
581nm	585 / 15	586 / 15	PE
	610 / 20	610 / 20	PE-Dazzle594
	670 / 30	670 / 30	-
		710 / 50	-
	780 / 60	770 / 40	PECy7
640nm	670 / 30	670 / 30	APC
	730 / 35	730 / 45	APC-R700
	780 / 60	770 / 40	APCFire-750

Each cytometer was individually QC'ed using different lots of CS&T beads, and 8-peak beads. There was no cross-calibration of the cytometers.

2 Supplementary figures

Figure S1 Example graphical output from DR (a) and clustering analysis (b) in selected non-command line platforms. (a) DR embedding of a single fcs file (donor- Spleen 423C) from FlowAtlas, FlowJo v 10.8.1 tSNE, and FCSExpress tSNE, coloured by CD3/IgD expression. Embedding scales and heatmap scales are not comparable between plots. (b) Example clustering results from FlowAtlas, FlowJo EmbedSOM plugin and Cytobank applied to a 23-colour spectral cytometry FCS file. Cytobank- inset shows the structure of the resulting minimum spanning tree (MST), which describes relationships between clusters and is coloured by CD56 MFI in this example.

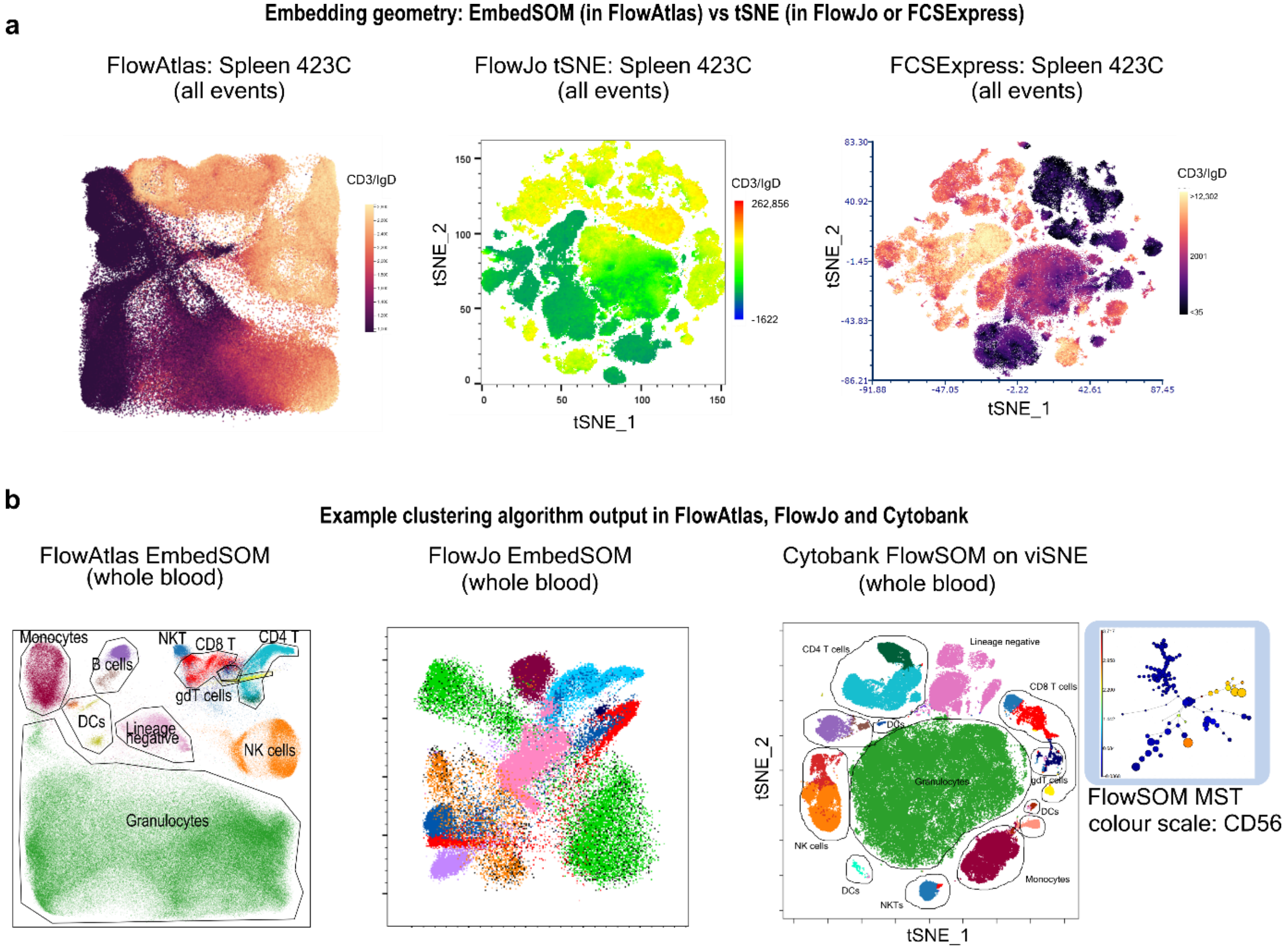

Figure S2 Gating strategy for 23-colour spectral cytometry panel of whole human blood. Debris, dead cells and doublets had already been excluded. Data from Cytobank experiment number 191382 were provided unmixed and compensated. This dataset was used to compare FlowAtlas computational performance at clustering and the workflow for rare population discovery versus Cytobank.

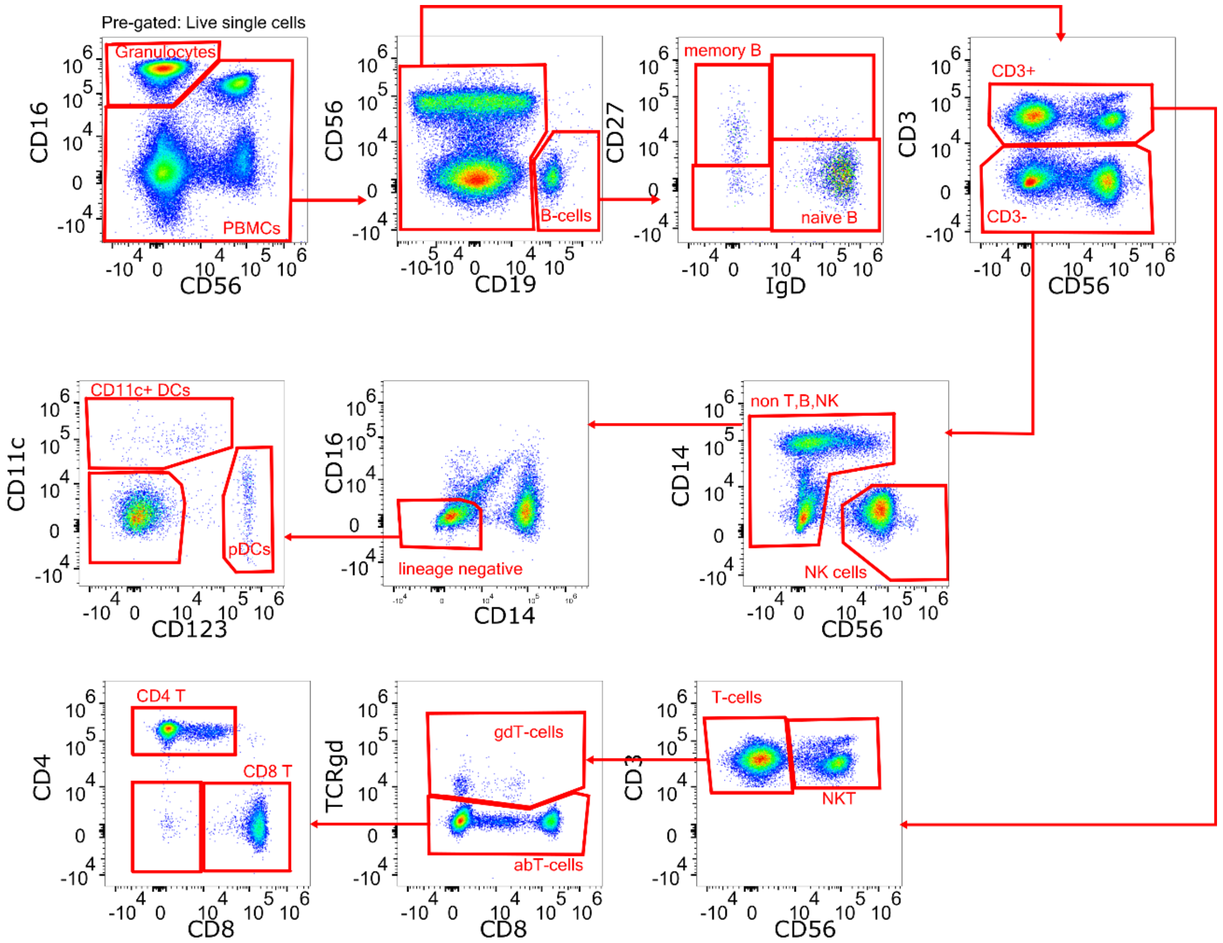

Figure S3 Stress testing FlowAtlas with incrementally larger and more complex datasets. Configuration used: Windows 11 OS 64 bit, 64 GB RAM, i7-13700H processor. Total event number and number of parameters determine computation time. Number of cells per FCS file is irrelevant.

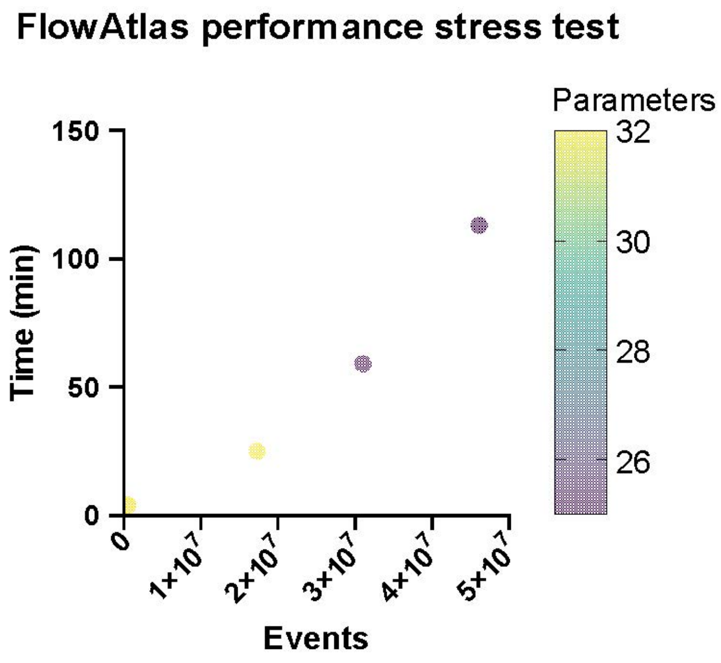

Figure S4 Exploration of Th1 memory cell subsets across tissues and donors in FlowAtlas. (a) Th1 cells were filtered in FlowAtlas, showing all donors coloured by tissue group, and 4 ROIs were drawn. Some tissues were coloured together due to low cell number per sample. (Spleen- cyan; bone marrow- gray; lymph nodes- yellow; non-lymphoid tissues- purple; blood- red) (b) Violin plots generated from the 4 ROIs in FlowAtlas. Th1 subsets differed by their expression of CD127, CD69 and PD-1. (c) Th1 cells with the superimposed ROIs, filtered by tissue type, confirmed that CD69+ cells are mainly found in lymphoid and non-lymphoid tissues; the non-lymphoid tissues contained a HLADR+PD1+ population (gray ROI) not present in other tissues. These markers may reflect past cell activation history. (d) The new Th1 subsets were validated in FlowJo by creating CD127/CD69 gates in all samples. (e) The updated FlowJo workspace was reopened in FlowAtlas to display 3 of the new Th1 suopulations. The HLADR+ PD1+ suopulation is hidden (it is a subset of CD127-CD69+ cells and was not gated in FlowJo; we confirmed it was only present in non-lymphoid tissues).

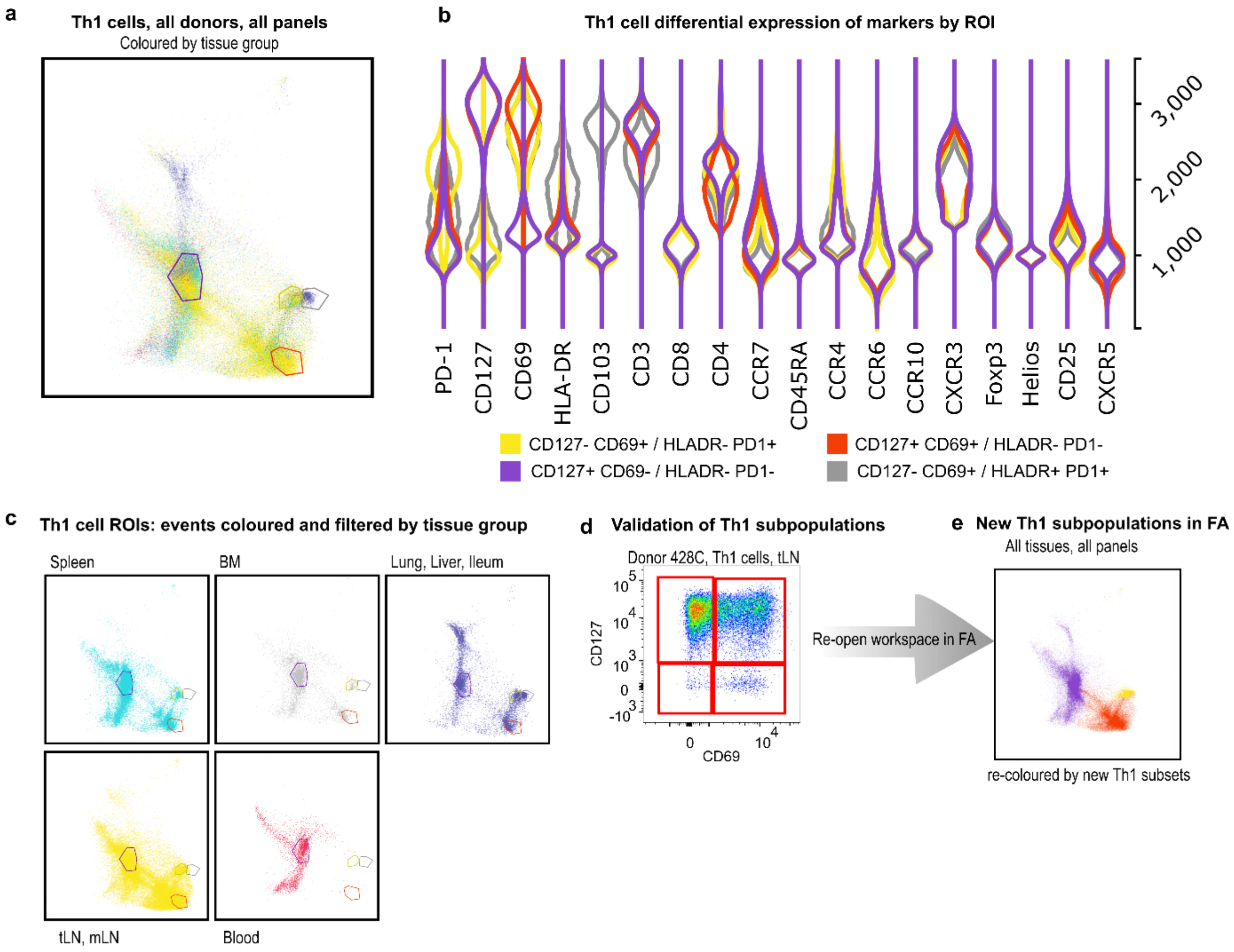

Figure S5 Exploratory analysis of the CD8⁺ effector-memory T cell population. (a) FlowAtlas-generated embedding of CD8⁺ T effector memory (EM) subset, as defined by the initial FlowJo gating strategy, displayed as a composite of all tissues, stained with all panels, and as tissue-specific embeddings. (b) ROIs were drawn around four of many possible subclusters in the composite embedding to auto-generate subcluster-specific violin plots of marker expression. CD69⁺ cells, indicated in cyan and yellow, were largely absent from blood but present in tissues. Although few cells were obtained from the ileum, nearly all co-expressed CD69 and the integrin CD103 as has been described(22),(23). Heatmaps of CD103 and CD69 expressions are shown on composite embeddings.

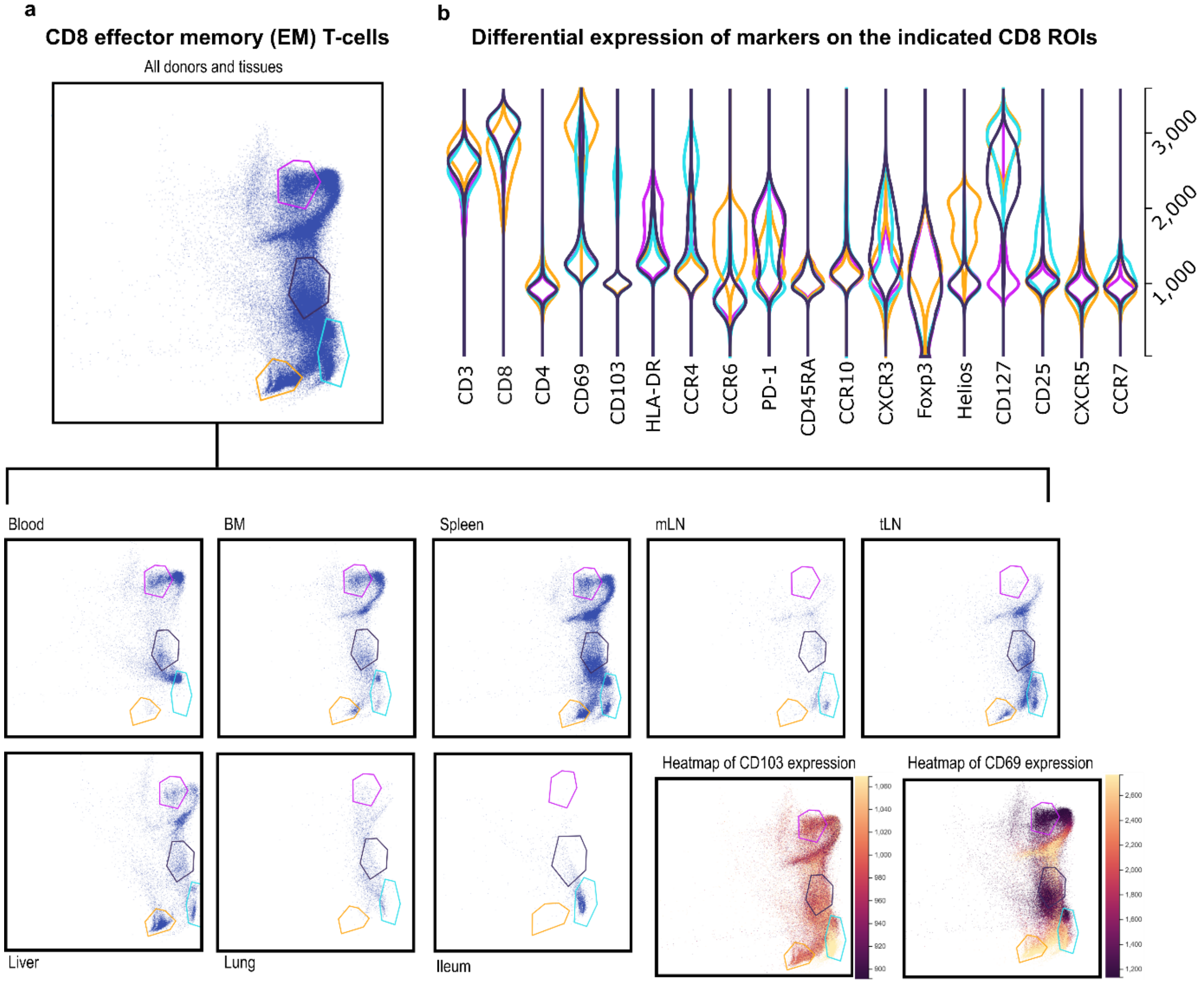

Figure S6 Relative abundance box plots of main T-cell populations in all donors and tissues calculated in FlowAtlas. Blood was available only for donors 412C and 423C. Percent of any combination of populations can be calculated, relative to their sum total. Although not shown, this includes populations which do not share a parent or grandparent gate, such as T-cells and B-cells. CD4 T-cell subsets, especially CM and naïve, were enriched in lymph nodes, whereas ileum, liver, and lung were dominated by CD8 cells, particularly EM and TEMRAs. Within the CD4 memory compartment, frequencies of T helper cell subsets were variable across donors and tissues, with Th2 cells being most frequent in blood. CM - central memory, EM -effector memory; TEMRA - T effector memory cells re-expressing CD45RA.

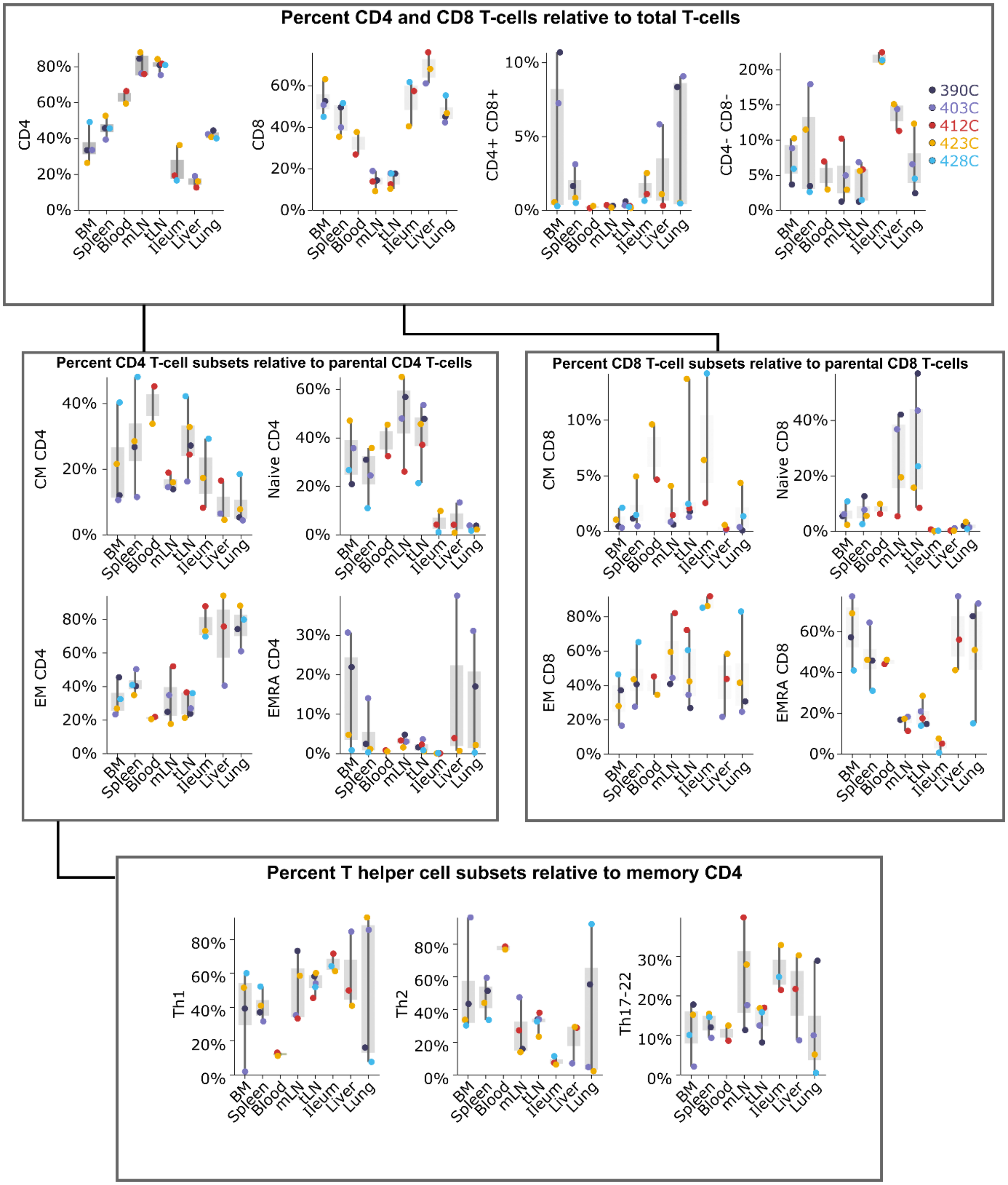

Figure S7 Example analysis of CyTOF data in FlowAtlas. (a) Data cleanup to remove irrelevant events, and transformation in FlowJo. (b) Gating strategy for population identification. (c) Embedding in FlowAtlas of exported clean FCS files; inset below- the same embedding coloured by 4 of the markers on the panel. (d) Violin plots of differential expression of markers from two regions in the embedding. Data source: FlowRepository FR-FCM-ZZNV, manuscript 27933748 (Yann Abraham et al, https://pubmed.ncbi.nlm.nih.gov/27933748/).

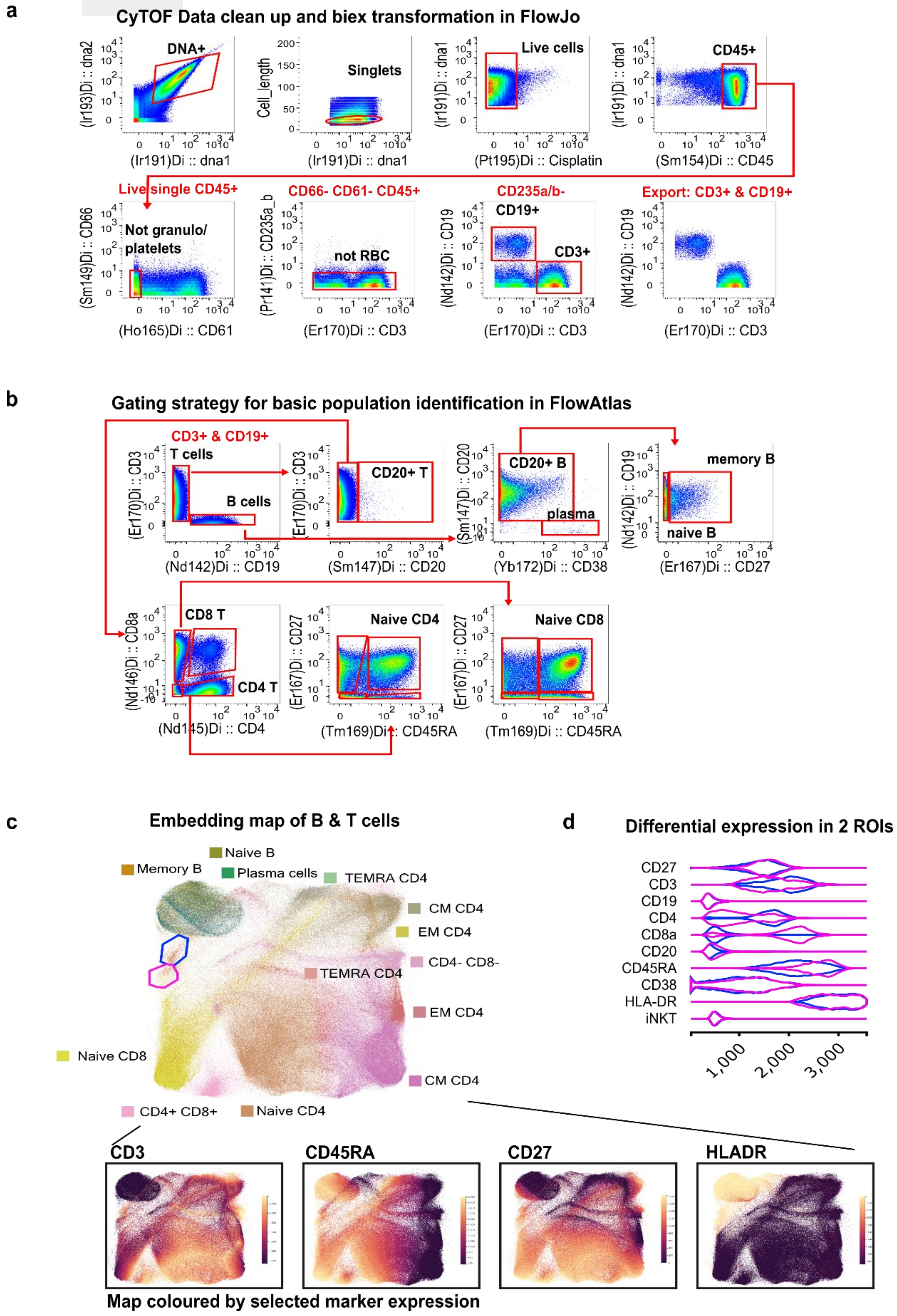

Figure S8 Failure of integration of two highly dissimilar panels due to an insufficient number of overlapping markers and/or fluorochromes. The same donor healthy blood sample was stained during a single experiment with the two indicated panels, using an identical procedure. Data were acquired on the same cytometer with identical settings on the same day. FCS files were cleaned of doublets, dead cells and debris, fully compensated and appropriately transformed prior to embedding. Markers and fluorochromes shared between the panels are indicated in bold.

	Marker	Panel 1	Panel 2
	**CD3**	**BUV737**	**BUV737**
	**CD4**	BUV395	Alexa Fluor 700
	CD8	BV650	-
	CD56	BV605	-
	CD19	PECy7	-
	CD16	Alexa Fluor 700	-
	**CD127**	FITC	PECy7
	**CD45RA**	**BV786**	**BV786**
	**CD27**	**V500**	**V500**
	**CD25**	PE	BB515
	CD122	BV421	-
	CR2 (CD21)	-	PECy5
	CD31	-	BV421
	Ki67	-	BUV395
	**Foxp3**	Alexa Fluor 647	PE-Dazzle 594
	Helios	PerCPCy5.5	
Total shared	7	3	3
Total unique	9	10	7
Total	16	13	10

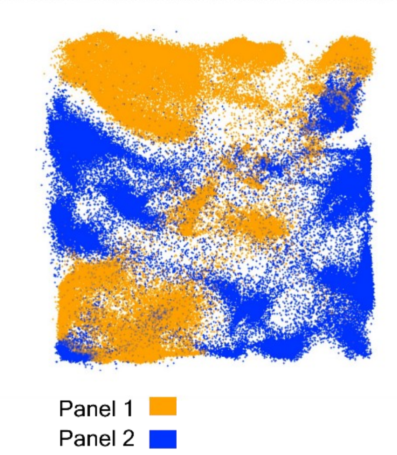

Figure S9 Computational correction of batch effects using CytoNorm before analysis in FlowAtlas. Batch effects arose from using antibodies from different manufacturing lots, causing non-biological differences in fluorescence intensity- and therefore in FlowAtlas embedding geometry. An inter-run control sample was stained alongside each experiment. This was used as a reference to correct the batch effects using the CytoNorm algorithm. (a) Left- uncorrected data and right- data corrected with CytoNorm, both displayed in FlowAtlas and coloured by batch. (b) Grouping variables in FlowAtlas for this demonstration experiment; (c) Scatter plots shows that these batch effects arose due to differences in CD3 and CD122 staining; left-before and right- after correction.

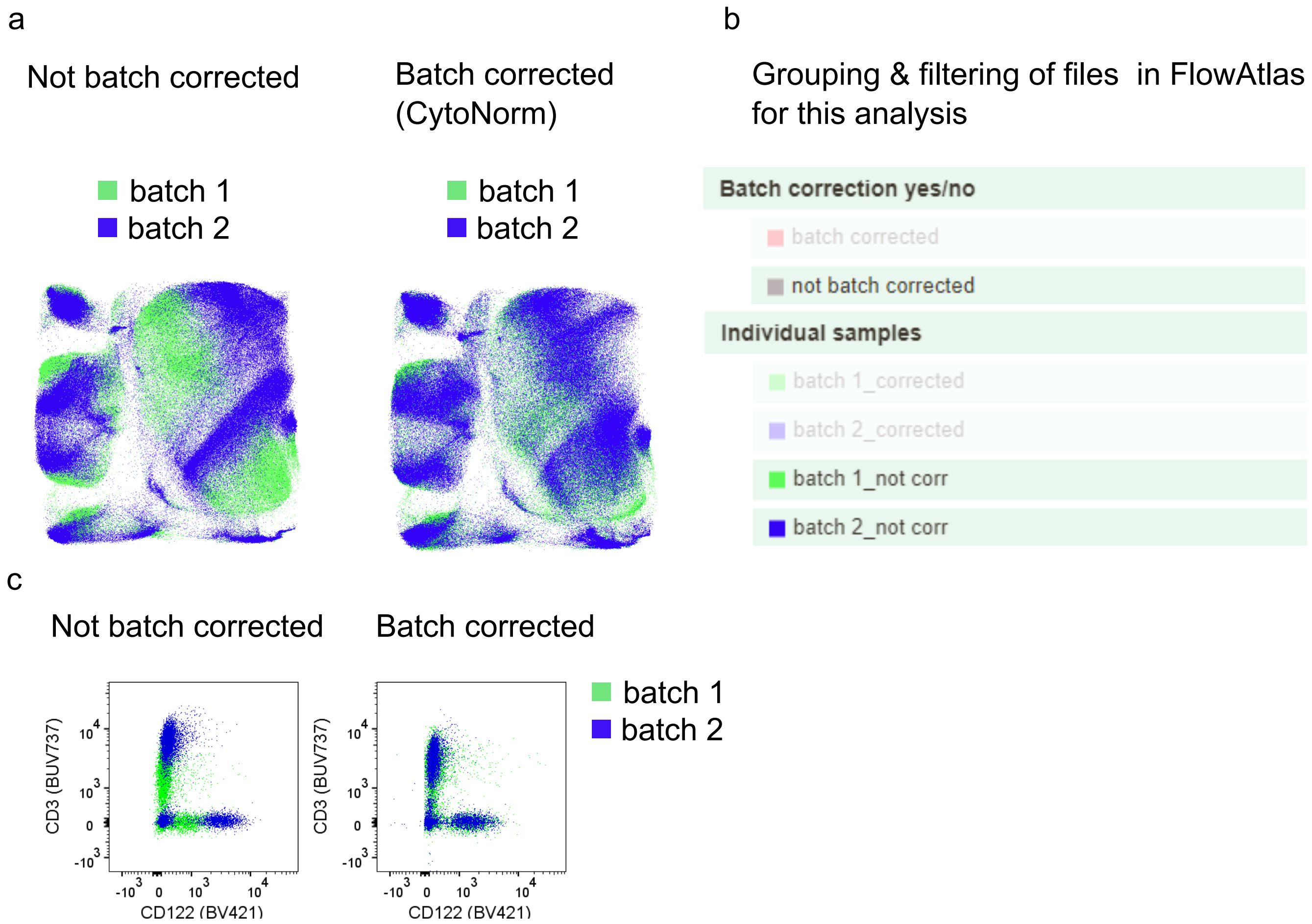

Figure S10 Overview of samples and data sources in this work. Figure generated with BioRender.

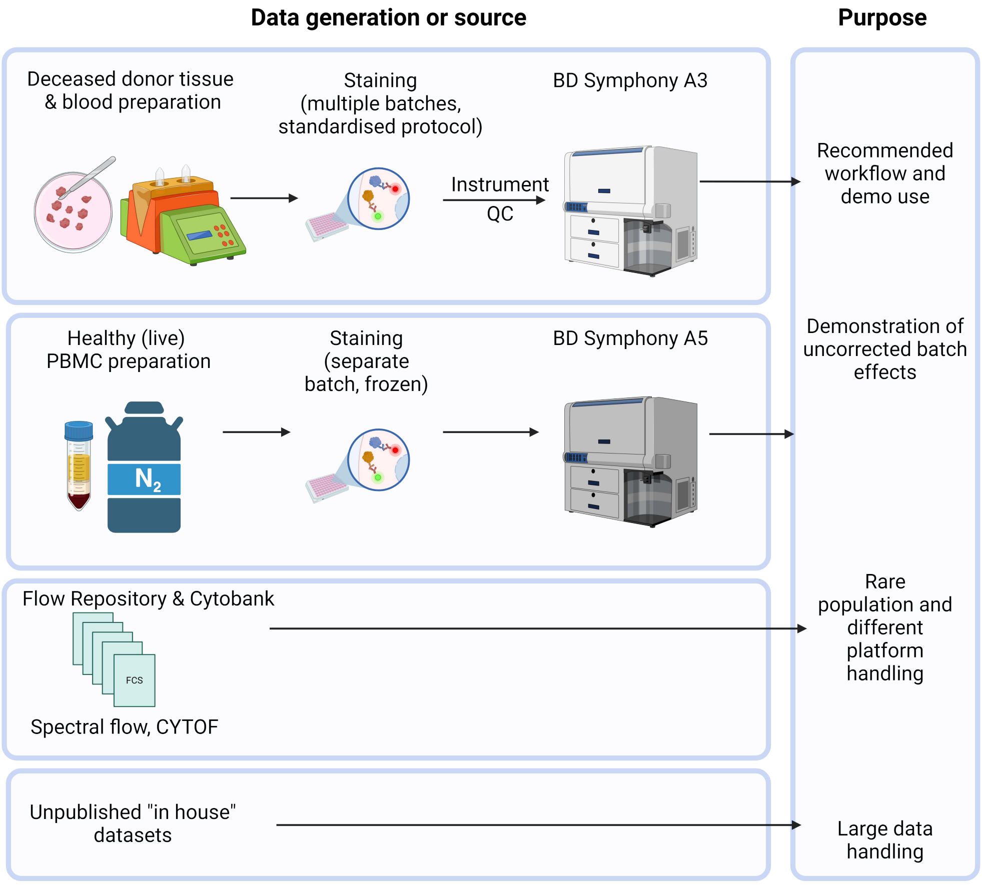

Supplement: Supplementary file 1 [file DataSheet_1.pdf]
